# Dissecting central post-stroke pain: a controlled symptom-psychophysical characterization

**DOI:** 10.1093/braincomms/fcac090

**Published:** 2022-04-05

**Authors:** Luciana Mendonça Barbosa, Valquíria Aparecida da Silva, Antônia Lilian de Lima Rodrigues, Diego Toledo Reis Mendes Fernandes, Rogério Adas Ayres de Oliveira, Ricardo Galhardoni, Lin Tchia Yeng, Jefferson Rosi Junior, Adriana Bastos Conforto, Leandro Tavares Lucato, Marcelo Delboni Lemos, Roland Peyron, Luis Garcia-Larrea, Manoel Jacobsen Teixeira, Daniel Ciampi de Andrade

**Affiliations:** Pain Center, Discipline of Neurosurgery HC-FMUSP, LIM-62, University of São Paulo, São Paulo, Brazil; Pain Center, Discipline of Neurosurgery HC-FMUSP, LIM-62, University of São Paulo, São Paulo, Brazil; Pain Center, Discipline of Neurosurgery HC-FMUSP, LIM-62, University of São Paulo, São Paulo, Brazil; Pain Center, Discipline of Neurosurgery HC-FMUSP, LIM-62, University of São Paulo, São Paulo, Brazil; Pain Center, Discipline of Neurosurgery HC-FMUSP, LIM-62, University of São Paulo, São Paulo, Brazil; Pain Center, Discipline of Neurosurgery HC-FMUSP, LIM-62, University of São Paulo, São Paulo, Brazil; Pain Center, Discipline of Neurosurgery HC-FMUSP, LIM-62, University of São Paulo, São Paulo, Brazil; Pain Center, Discipline of Neurosurgery HC-FMUSP, LIM-62, University of São Paulo, São Paulo, Brazil; Department of Neurology, LIM-62, University of São Paulo, 05403-900 São Paulo, Brazil; Department of Radiology, LIM-44, University of São Paulo, 05403-900 São Paulo, Brazil; Department of Radiology, LIM-44, University of São Paulo, 05403-900 São Paulo, Brazil; NeuroPain Team, Lyon Neuroscience Research Center (CRNL), Inserm U1028, CNRS UMR5292, UCBL1, UJM, F-6900 Lyon, France; NeuroPain Team, Lyon Neuroscience Research Center (CRNL), Inserm U1028, CNRS UMR5292, UCBL1, UJM, F-6900 Lyon, France; Pain Center, Discipline of Neurosurgery HC-FMUSP, LIM-62, University of São Paulo, São Paulo, Brazil; Department of Neurology, LIM-62, University of São Paulo, 05403-900 São Paulo, Brazil; Pain Center, Discipline of Neurosurgery HC-FMUSP, LIM-62, University of São Paulo, São Paulo, Brazil; Center for Neuroplasticity and Pain, Department of Health Sciences and Technology, Faculty of Medicine, Aalborg University, DK-9220 Aalborg, Denmark

**Keywords:** central post-stroke pain, post-stroke pain, neuropathic pain phenotyping, central neuropathic pain, quantitative sensory testing

## Abstract

Central post-stroke pain affects up to 12% of stroke survivors and is notoriously refractory to treatment. However, stroke patients often suffer from other types of pain of non-neuropathic nature (musculoskeletal, inflammatory, complex regional) and no head-to-head comparison of their respective clinical and somatosensory profiles has been performed so far. We compared 39 patients with definite central neuropathic post-stroke pain with two matched control groups: 32 patients with exclusively non-neuropathic pain developed after stroke and 31 stroke patients not complaining of pain. Patients underwent deep phenotyping via a comprehensive assessment including clinical exam, questionnaires and quantitative sensory testing to dissect central post-stroke pain from chronic pain in general and stroke. While central post-stroke pain was mostly located in the face and limbs, non-neuropathic pain was predominantly axial and located in neck, shoulders and knees (*P* < 0.05). Neuropathic Pain Symptom Inventory clusters burning (82.1%, *n* = 32, *P* < 0.001), tingling (66.7%, *n* = 26, *P* < 0.001) and evoked by cold (64.1%, *n* = 25, *P* < 0.001) occurred more frequently in central post-stroke pain. Hyperpathia, thermal and mechanical allodynia also occurred more commonly in this group (*P* < 0.001), which also presented higher levels of deafferentation (*P* < 0.012) with more asymmetric cold and warm detection thresholds compared with controls. In particular, cold hypoesthesia (considered when the threshold of the affected side was <41% of the contralateral threshold) odds ratio (OR) was 12 (95% CI: 3.8–41.6) for neuropathic pain. Additionally, cold detection threshold/warm detection threshold ratio correlated with the presence of neuropathic pain (*ρ* = −0.4, *P* < 0.001). Correlations were found between specific neuropathic pain symptom clusters and quantitative sensory testing: paroxysmal pain with cold (*ρ* = −0.4; *P* = 0.008) and heat pain thresholds (*ρ* = 0.5; *P* = 0.003), burning pain with mechanical detection (*ρ* = −0.4; *P* = 0.015) and mechanical pain thresholds (*ρ* = −0.4, *P* < 0.013), evoked pain with mechanical pain threshold (*ρ* = −0.3; *P* = 0.047). Logistic regression showed that the combination of cold hypoesthesia on quantitative sensory testing, the Neuropathic Pain Symptom Inventory, and the allodynia intensity on bedside examination explained 77% of the occurrence of neuropathic pain. These findings provide insights into the clinical-psychophysics relationships in central post-stroke pain and may assist more precise distinction of neuropathic from non-neuropathic post-stroke pain in clinical practice and in future trials.

## Teaser

Dissection of distinct post-stroke pain syndromes evidenced that the neuropathic pain inventory, the presence of cold thermal deficit and the finding of allodynia on bedside examination, explained 77% of the occurrence of neuropathic central post-stroke pain, a new finding that has clear diagnostic potential.

## Graphical Abstract

**Graphical Abstract:**
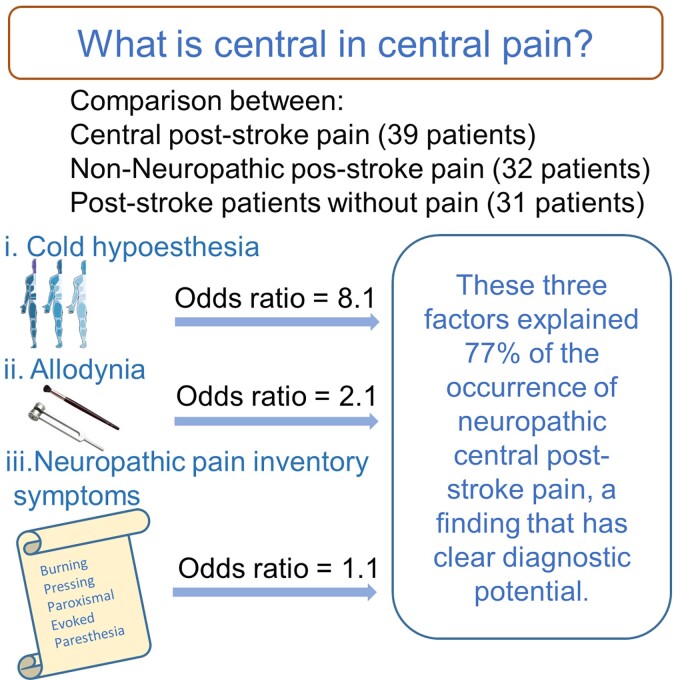

## Introduction

Along with motor, language and coordination deficits, stroke may also lead to pain in up to 50% of individuals.^1–4^ Post-stroke pain (PSP) includes several different pain syndromes such as musculoskeletal, spasticity-related, headaches, complex regional pain syndrome and central neuropathic pain (i.e. central post-stroke pain—CPSP).^2,5^ CPSP occurs in 1–12% of stroke patients in general and is highly refractory to treatments.^5^ Indeed, a number of medications^6^ and neuromodulatory approaches^7,8^ that have been shown to relieve pain in peripheral neuropathic pain^9,10^ have failed to do so in CPSP,^11^ the mechanisms of which are poorly understood. Although significant insights have been gained from neuroimaging,^12–18^ neurophysiology,^19–23^ basic studies^24–28^ and psychophysics,^18,29–39^ currently no efforts have been made to integrate a comprehensive clinical characterization of these patients with the concomitant abnormalities of the somatosensory system in a controlled fashion that includes PSP of non-neuropathic origin, by far the most common PSP subtype.

Additionally, sensory abnormalities in neuropathic pain reflect altered mechanisms of nociceptive processing, and neuropathic pain mechanisms are thought to be diverse within a single disease aetiology.^40^ So, efforts to define the optimal method to classify patients based on symptoms profile, bedside examination and quantitative sensory testing (QST)^41^ have been made in an attempt not only to diagnose neuropathic pain itself, but also to stratify patients who are more likely to respond to specific therapeutic interventions.^41–47^ By doing so, one expects to replace the current treatment strategies proposing to treat all patients similarly, which has provided relatively poor symptomatic control.^48,49^ For this aim, studies exploring the relationship between symptoms and somatosensory loss and gain of function are required. To date, studies exploring symptom-psychophysics relationships have included mainly patients with peripheral neuropathic pain,^40,50^ while symptom-psychophysics correlations in CPSP remain less common, often without standardized symptom characterization^51^ and with either no control groups or one composed of patients without chronic pain. In order to dissect CPSP from PSP in general, we have compared a sample of CPSP patients with non-neuropathic PSP patients and with stroke patients without chronic pain matched for sex, age and stroke location. We have evaluated stroke characteristics, neuropathic pain symptoms, bedside examination and static and dynamic QST across groups to provide a deep phenotyping of CPSP and describe potential symptom-QST correlations specific to CPSP that could be used in the future in preventive or therapeutic trials.

## Materials and methods

This was a controlled cross-sectional study, part of the Central Pain Initiative Project focused on the assessment and treatment of central neuropathic pain.^11^ The present study aimed to compare pain characteristics and sensory profile of CPSPs with two control groups: (i) patients with non-neuropathic PSP (PSP-Non) and (ii) stroke patients without chronic pain (No-Pain). The three groups were matched according to sex, age and stroke area.

### Standard protocol approvals and patient consent

Data collection took place at the Hospital das Clínicas, Faculdade de Medicina da Universidade de São Paulo (HC-FMUSP). It was approved by the Institution’s Ethics Review Board (No. 690.455). All patients were volunteers and informed about the procedures and provided written informed consent before inclusion in the study. No financial compensation was offered for study participation. Neuroimaging findings form part of these patients have been reported elsewhere.^52^

### Patients

**Figure 1:**
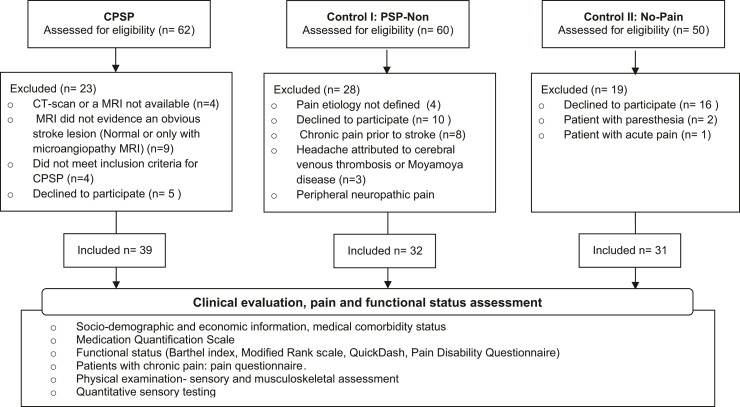
**STROBE flow diagram of patient recruitment according to pain characteristics.** CPSP, central post-stroke pain; PSP-Non, non-neuropathic post-stroke pain; No-Pain, stroke patients without chronic pain.

According to clinical evaluation and imaging information, two neurologists trained in pain management and one neuroradiologist (L.M.B., J.R.J. and L.T.L.) classified each patient’s pain syndrome. All cases were confirmed by a board (D.C.d.A. and M.J.T.), and only patients with consensual pain classifications were included. All participants had suffered an ischaemic or haemorrhagic stroke at least 3 months before the evaluation was confirmed by imaging (CT or MRI). Exclusion criteria were major cognitive or language impairments that would compromise filling in questionnaires or sensory examination (Fig. 1). Also, patients with more than one stroke needed to have deficits related to only one of the strokes, with a normal examination otherwise (i.e. unilateral deficits). This granted that mirror areas had no sensory deficits due to previous strokes. The CPSP sample was composed of patients consecutively referred to the pain centre by neurologists or primary care physicians, and fulfilling the following criteria: (i) definite diagnosis of neuropathic pain according to the NeuPSIG/International Association for the Study of Pain (IASP) (IASP Special Interest Group on Neuropathic Pain) grading system for neuropathic pain^53^; (ii) occurrence of *de novo* pain attributed to a central lesion due to stroke; (iii) pain characteristics not compatible with other aetiologies of pain (previous fibromyalgia, migraine, nociceptive pain).^5^

#### Control groups

CPSP patients were compared with two control groups: (i) PSP that was non-neuropathic in nature (PSP-Non) and (ii) No-Pain. These groups were recruited from the cerebrovascular diseases outpatient clinic from the Department of Neurology, University of São Paulo. They were matched according to sex, age and stroke location (i.e. divided into three macro-regions: cortical, subcortical and brainstem/cerebellum, by a blinded neuroradiologist).^54^

##### The PSP-Non group

Post-stroke painful symptoms present most of the days for longer than 3 months with a clear non-neuropathic aetiology (i.e. muscle spasms, spasticity, headache, musculoskeletal pain/myofascial pain syndrome, frozen shoulder), in the absence of concomitant neuropathic pain according to the IASP/NeuPSIG grading system. The presence of chronic pain *before* the stroke was an exclusion criterion for the PSP-Non group.^5^

##### The No-Pain group

Included patients without chronic pain before or after stroke, and no episode of acute pain (e.g. episodic headaches) within the 7 days preceding the clinical evaluation.

### Assessment

Participants were assessed in a single visit. They underwent a clinical evaluation, which included an analysis of current symptoms and limitations, and physical examination focused on sensory musculoskeletal systems. Sociodemographic information, medical comorbidity status and medication use were registered. Concomitantly, functional scores, questionnaires to evaluate pain, incapacity, mood and catastrophism, were also filled out as detailed below.

#### Functional assessment

The subsequent scales were employed to assess functional status:

- Barthel index^55,56^: Quantifies the level of independence, varies from 0 to 100 (100 is totally independent and 0 is entirely dependent on daily activities).
- Modified Rankin scale (mRS)^56,57^: Seven-point scale for functional outcome after stroke anchored at 0 = asymptomatic and 6 = death.
- Shortened disabilities of the arm, shoulder and hand questionnaire (QuickDash)^58^: Assesses disability, limitations for social activities and work, the severity of pain, and the interference with sleep, related to arm, shoulder and hand symptoms (i.e. a 100-point scale; the higher the score, the worse the upper limb disability.^59^
- Pain Disability Questionnaire (PDQ)^60,61^: It is composed of two factors: (i) functional status component (maximum score of 90) and (ii) psychosocial component (maximum score of 60). The total PDQ score consists of the sum of all items (maximum score of 150, with higher scores indicating more severe disability).

#### Pain scales and questionnaires

The following questionnaires were used to assess pain in the CPSP and PSP-Non groups:

- Short-form McGill Pain Questionnaire (SF-MPQ): It has pain descriptors divided into three dimensions: sensory (eight items), affective (five items) and evaluative (two items).^62^ Sensory, affective, evaluative and total descriptors are obtained by counting the words chosen by the patient.^62,63^
- Brief Pain Inventory: Measures pain intensity (least, average, now and worst pain in the last 24 h, each ranging from 0—no pain to 10—maximal pain imaginable); and interference scores (general activity, mood, walking ability, normal work, relationships with others, sleep and enjoyment of life, with a total score ranging from 0 to 70, where higher scores mean higher inference of pain in daily activities).^64,65^
- Douleur Neuropathique Questionnaire-4 (DN-4): A screening test for neuropathic pain composed of ten items. It ranges from 0 to 10 and is positive when ≥4.^66,67^
- Neuropathic Pain Symptoms Inventory (NPSI): A qualitative and quantitative inventory of different neuropathic pain descriptors that enables the evaluation of different phenotypes through discrimination and quantification of five distinct clinically relevant dimensions of neuropathic pain: burning (superficial) spontaneous pain, pressing (deep) spontaneous pain, paroxysmal pain, evoked pain and paresthesia/dysesthesia. Its total score ranges from 0 to 100, and each dimension’s score ranges from 0 to 10 with higher scores indicating more intense symptoms.^68,69^ Recently, a new cluster has been proposed, classifying patients into three groups according to an artificial intelligence algorithm applied to the scores of each of the items: pinpointed, deep and provoked pain.^41^ Cut-off point of NPSI total score differentiating neuropathic from non-neuropathic pain was assessed through receiver operating characteristic (ROC) curve analysis.

#### Quality of life, mood and catastrophism assessment

- The Short Form 12-Health Status Questionnaire (SF-12): Measures health-related quality of life and is composed of 12 items that generate two scores related to physical health (PCS) and mental health (MCS). Each score ranges from 0 to 100; the higher the score, the greater the health-related quality of life.^70^
- Hospital Anxiety and Depression Scale: Evaluates symptoms of anxiety and depression; higher scores mean more severe symptoms. Scores of eight for anxiety and nine for depression were used as cut-off values.^71,72^
- The Pain Catastrophization Scale: Assesses catastrophic thoughts or feelings accompanying the experience of pain. This scale consists of 13 items on a Likert scale. The total scale score ranges from 0 to 52; higher scores represent greater catastrophic thinking.^73^

### Physical examination

#### Physical examination—musculoskeletal assessment

Spasticity in the upper and lower limbs was quantified according to the modified (m-) Ashworth spasticity scale (AS), in which higher values indicate more severe spasticity.^74^ It was classified into three categories—absent, low to moderate (m-AS 1 or 2 in at least one limb) and moderate to severe (m-AS score above 2 in at least one limb).^75^ Muscle strength was measured according to the Medical Research Council (MRC) scoring system. Motor impairment degree was grouped into four severity grades—Grade 0 (MRC in all limb = 5), Grade 1 (MRC = 4 in at least one limb), Grade 2 (MRC = 2 or 3 in at least one limb) and Grade 3 (MRC = 01 or 1 in at least one limb).^76^ Myofascial trigger points (TP) were evaluated bilaterally in standardized regions, including temporal, masseter, scalene, trapezius, pectoralis major, levator scapulae, rhomboid, supraspinatus, biceps brachii, triceps brachii, wrist and finger extensors, first dorsal interosseous, quadratus lumborum, gluteus maximus, piriformis, vastus lateralis and gastrocnemius muscles.^77^ Briefly, TP were looked for with 4 kg/cm^2^ of pressure, using the thumb (just enough to blanch the examiner’s nailbed).^78^ Active TP were considered to be present when digital pressure evoked pain in a corresponding referred pain pattern and resembled at least 50% of the patients’ clinical pain.^79,80^

#### Sensory assessment—bedside examination

The sensory assessment employed standardized bedside examination, including the evaluation of superficial touch and allodynia with a piece of cotton wool, cold sensitivity and cold allodynia with a metal rod at room temperature, and mechanical pain sensitivity by light prick with a pin. Regions of the face, trunk, arms and legs were tested, comparing them with the contralateral side and proximal and distal body regions.^79^ Hyperpathia was assessed with a pin: patients were asked to quantify the evoked pain during examination using the numeric rating scale (NRS: 0–10, where 0 means no pain and 10 maximal pain imaginable) after one stimulus and after a train of 10 stimuli delivered at 1 Hz.^81^ Allodynia intensity (NRS) differentiating neuropathic from non-neuropathic pain patients was assessed through ROC curve analysis.

### Static and dynamic QST

CPSP patients underwent a static QST battery to assess sensory findings at the site of the most severe neuropathic pain area (pain area) and the corresponding contralateral site (mirror area).^36,39,82^ PSP-Non and No-Pain groups were tested over the area of most severe motor/sensory abnormalities (contralateral to stroke) and its respective asymptomatic mirror area. If the patient presented bilateral symptoms, the worse area was tested, and the contralateral mirror area was used as the control side. In all areas, the following QST parameters were tested according to previously described techniques: briefly, cold detection threshold (CDT), warm detection threshold (WDT), mechanical detection threshold (MDT), vibration detection threshold (VDT), cold pain threshold (CPT), heat pain threshold (HPT), mechanical pain threshold (MPT) and the numerical pain rating scale for suprathreshold cold (STCP), suprathreshold heat pain (STHP), suprathreshold mechanical pain (STMP) and wind up ratio (WUR) were evaluated. The tests were assessed by the method of limits.^11,83^

Results were analysed in three outputs according to specific research questions:

- Side-to-side differences: Comparisons were made within-subjects (pain or affected area versus mirror area^18,31,34,36,37,39,84–86^), and a QST index of asymmetry was obtained to assess differences between groups so that threshold or hyperalgesia indices for each QST parameter were calculated according to the formula: value from the test area/value from the mirror area.
- Single-patient classification: Single QST results from each parameter were classified as normal or abnormal according to Rolke *et al*.^87^ recommendations for side-to-side comparisons, so that a ratio (values from tested area/values from the mirror area) was calculated and considered abnormal if it was above or below the following lower and upper cut-off values for: CDT (0.41–2.42), WDT (0.42–2.39), MDT (0.38–2.62), MPT (0.4–2.53), WUR (0.52–1.94).^87^ For CPT and HPT, the difference between results from test and mirror areas (test area–mirror area) was calculated and considered abnormal if it was above or below the following lower and upper cut-off values: CPT (−10.3 to 10.3°C) and for HPT (−4.2 to 4.2°C).^87^ For VDT, STCP, STHP and STMP, abnormal values were considered for indices below 0.4 or above 2.5.
- Thermal limen assessment: Since warm and CDTs were the sensory modalities reported to be more starkly altered in CPSP,^18,31,34,36,37,39,84,85^ a ‘thermal ratio’ was created consisting of CDT pain area × WDT mirror/CDT mirror × WDT pain area. This CDT/WDT thermal ratio is analogous to the ‘sensory limen’^83^ or the sensitivity index proposed by Jensen *et al*.^88^ and used by Vestergaard *et al*.^84^. It was intended to illustrate unbalance between cold and WDTs (something that has been associated with experimental allodynia under the thermal grill illusion of pain and in spinal cord injury patients^89,90^).

Pressure pain threshold (PPT) was assessed with an algometer (Pain Diagnostics & Thermograph Inc., Great Neck, NY, USA) in patients with chronic pain in the same muscles tested in the myofascial pain investigation, as described above. The rubber tip of the algometer was vertically positioned on the point to be examined. The pressure was increased at ∼1 kg/s continuously until the subject reported the triggering of pain or discomfort. The lowest pressure value-generating pain at each point was considered as the PPT.^91^ Furthermore, the deep pressure hyperalgesia [i.e. the intensity of the pain (0–10 NRS)] generated by a three second-stimulation at the PPT + 2 kg/cm^2^ was also measured for each muscle site.^92^

Dynamic QST was assessed by conditioned pain modulation (CPM) and was evaluated by measuring the pain intensity of a stimulus (test-stimulus—suprathreshold heat pain stimulus over the thigh not affected by stroke) which was then repeated after a painful tonic stimulus (conditioning cold pressor test—immersion of the contralateral hand).^93,94^ CPM was reported as the evoked pain intensity difference between the conditioned and unconditioned test stimuli.

### Statistical analysis

Categorical variables were represented by frequencies, percentages and absolute numbers. Quantitative variables were tested for normal distribution using Kolmogorov–Smirnoff tests and Q–Q plots and histograms. The Kruskal–Wallis test was employed for comparisons of non-parametric quantitative variables between the three groups. The Mann–Whitney test was applied for comparisons of non-parametric quantitative variables between two groups, and the Bonferroni correction was used for multiple comparisons. The *χ*^2^ test and Fisher’s exact test were used to compare the nominal and ordinal qualitative variables between groups. OR and 95% confidence intervals (CIs) were calculated to assess the relations between neuropathic pain and somatosensory abnormalities assessed through physical examination and QST. Spearman coefficients were used to assess the correlation between variables found to be significantly different. Correlations with a correlation coefficient ≥ ± 0.4 were included in logistic regression analyses. Basic assumptions including independence of errors, linearity in the logit for continuous variables, absence of multicollinearity and lack of strongly influential outliers were checked before the test. The study size was estimated based on the proportion of the most prominent finding on QST in CPSP (mechanical allodynia) according to one of the largest studies to date.^86^ This was a convenience sample with 31 patients in the smallest group allowing to detect a difference in proportion around 23% between chronic pain groups with a power of 80% and a Type I error set at 5% bilaterally. The estimated sample size was also in line with the CPSP sample size of previous studies.^31,34,36,37,82,95–98^ The level of significance considered was 5%. Since it was an exploratory study, adjusting for multiple testing was not mandatory. However, we opted to evaluate the subgroup analysis with a pairwise correction to distill the more robust findings that could be inputed into the regression model,^99^ so Bonferroni correction for multiple testing was performed when indicated.

### Data availability

The data that support the findings of this study are available on request from the corresponding author.

## Results

### Sample characteristics

A total of 102 stroke patients were evaluated; 39 had central neuropathic pain due to stroke (CPSP group), 32 patients had chronic pain of non-neuropathic origin with onset post-stroke–(PSP-Non group) and 31 were pain-free (No-Pain) (Fig. 1). The mean age was 59.4 (±11.9) years, with no significant differences among groups (*P* = 0.28). Male patients made up 64.7% of the total sample, and most medical comorbidities were similar between groups (Supplementary Tables 1 and 2).

### Stroke characteristics

The stroke location (cortical, 38.2%; subcortical, 37.3%; brain stem/cerebellum, 24.5%), the time elapsed since stroke (47.7 ± 44.3 months), the type of event (ischaemic 86.1%, haemorrhagic 13.9%) and the number of lesions (20.6% had more than one) were distributed similarly in the three groups, with no significant differences among them (Supplementary Table 3).

### Pain characteristics

**Figure 2:**
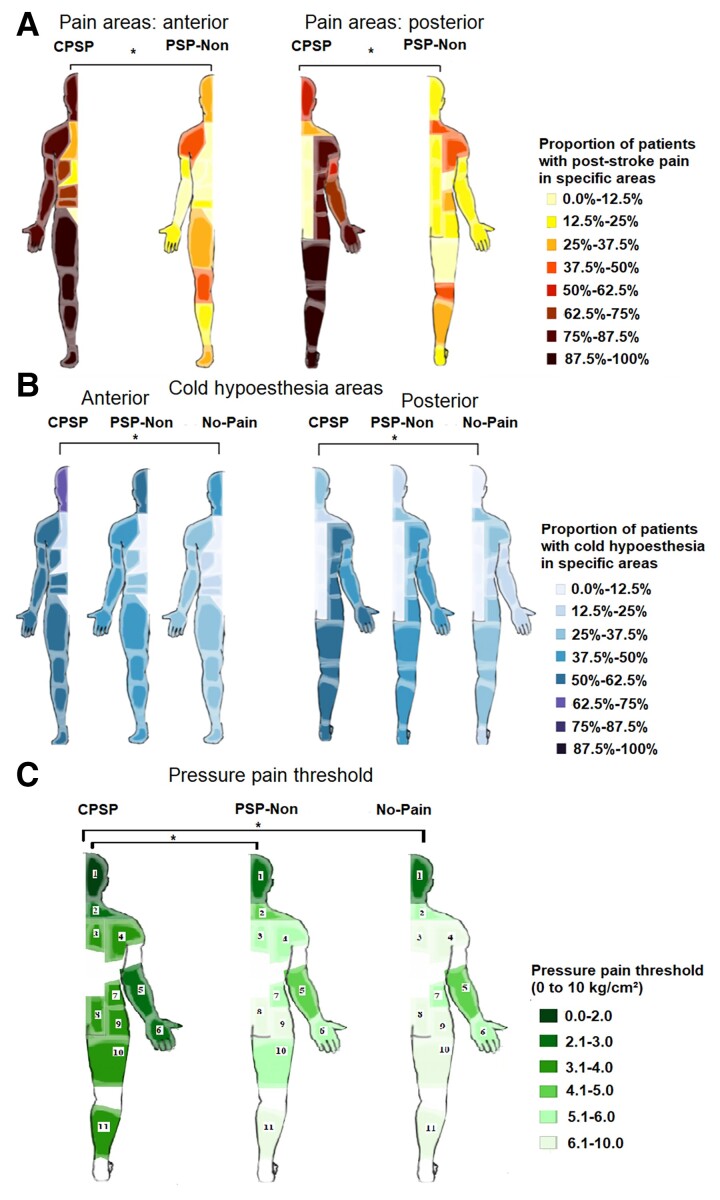
**Pain area and cold hypoesthesia distribution according to pain groups frequency.** (**A**) Pain area distribution according to pain groups.*P was <0.05 for all areas except pelvic and lumbar regions. (**B**) Cold hypoesthesia distribution. *P < 0.0167 (with Bonferroni correction for multiple comparisons). (**C**) PPT *P < 0.0167 (with Bonferroni correction for multiple comparison). Tested areas: 1, temporal and masseter; 2, trapezius; 3, rhomboid; 4, levator scapulae, supraspinatus; 5, wrist and finger extensors; 6, first dorsal interosseous; 7, quadratus lumborum; 8, gluteus maximus; 9, piriformis; 10, vastus lateralis; 11, gastrocnemius. CPSP, central post-stroke pain; PSP-Non, non-neuropathic post-stroke pain; No-Pain, stroke without pain.

The mean duration of pain was 47.3 (±47.2) months without difference between groups (*P* = 0.949). CPSP pain was mainly located in the face, upper and lower limbs (Fig. 2); 79.5% of CPSP patients (*n* = 31) considered their pain as continuous compared with 40.6% in the PSP-Non group (*n* = 13), *P* = 0.001. Pain in the PSP-Non pain group was mainly axially located: in the neck, shoulders and knees (Fig. 2). Pain occurred within body areas presenting sensory abnormalities confirmed on physical examination in 100% of CPSP patients and 37.5% of PSP-Non patients (*P* < 0.001). The spatial distribution pattern of pain areas in this subgroup of PSP-Non patients was similar to the rest of the PSP-Non group (Supplementary Fig. 1). In all cases, these patients had a clear aetiology for their pain as of non-neuropathic origin (i.e. osteoarthritis, spasticity, tendinitis or bursitis) and a negative DN-4. The most common pain type in the non-neuropathic pain group was musculoskeletal pain. Exclusive musculoskeletal pain made up 68.8% (*n* = 22), chronic headache (more than 15 days per month for 3 months), 12.5% (*n* = 4) and headache associated with musculoskeletal pain 18.8% (*n* = 6) of this group.

### Pain assessment

CPSP patients had significantly higher scores for the intensity of both sensory (5.7 ± 1.7 versus 3.5 ± 2.0, *P* < 0.001) and affective dimensions of pain (3.9 ± 1.4 versus 2.9 ± 1.4, *P* = 0.003) (Supplementary Tables  4 and 5). The specific pain symptoms most frequently reported in the CPSP group were burning (82.1%, *n* = 32, *P* < 0.001), tingling (66.7%, *n* = 26, *P* < 0.001) and pain evoked by cold stimulus (64.1%, *n* = 25, *P* < 0.001). PSP-Non patients never described their pain as tingling or as electric shocks. The pain descriptors were clustered in the five distinct dimensions of neuropathic pain, and the burning (superficial) spontaneous pain dimension corresponded to the highest scores in the CPSP. All scores, except the pressing (deep) spontaneous pain, were significantly higher in the CPSP group compared with the PSP-Non (Supplementary Fig. 4 and Table 6). Similar findings were found when cluster symptoms were classified according to Bouhassira *et al*.,^41^ where ‘provoked pain’ was more common in CPSP patients, ‘pinpointed pain’ occurred exclusively in CPSP, whereas ‘deep pain’ was more common in the non-neuropathic PSP-Non group. The NPSI total score cut-off point for neuropathic pain was 20/100, with a sensitivity of 87% and specificity of 28% (Supplementary Fig. 2).

### Quality of life, mood and function

Quality of life, mood and catastrophism ratings were worse in patients with chronic pain (both CPSP and PSP-Non) compared with No-Pain (Supplementary Table 7). The Barthel index revealed lower scores in CPSP groups, followed by PSP-Non, and No-Pain, *P* = 0.013. The mRS followed the same trend, with a higher concentration in mRS 3 and 5 in the CPSP (CPSP 35.8%, *n* = 14 versus PSP-Non 15.6%, *n* = 5, and versus No-Pain 16.1%, *n* = 5), *P* = 0.013. Comparisons between groups evidenced a difference when comparing CPSP versus No-Pain for the Barthel index (*P* = 0.004) and the mRS (*P* = 0.005)—Supplementary Table 8.

Upper limb disability was most prevalent in the CPSP group, followed by PSP-Non and No-Pain, *P* < 0.001. Pairwise comparisons confirmed differences between all pairs. A similar trend was observed for the PDQ (Supplementary Table 8).

### Physical examination

Thermal and dynamic mechanical allodynia was observed more frequently in CPSP [61.5% (*n* = 24) for both types] (Table 1), and both types of allodynia occurred concomitantly in 48.7% of CPSP patients. The allodynia NRS cut-off point for neuropathic pain was 2/10, with a sensitivity of 61% and specificity of 1.6% (Supplementary Fig. 3).

**Table 1:** Sensory assessment

	Group according to pain classification				
	CPSP *n* = 39	PSP-Non *n* = 32	No-Pain *n* = 31	Total *n* = 102	*P* effects between groups
*Physical examination—sensory testing*					
Tactile hypoesthesia	30 (78.9%)^a^	19 (59.4%)^a,b^	13 (41.9%)_b_	62 (61.4%)	0.007^*,†^
Cold hypoesthesia	24 (61.5%)	19 (59.4%)	13 (41.9%)	56 (54.9%)	0.217
Mechanical hypoalgesia	24 (61.5%)^a^	20 (62.5%)^a^	11(35.5%)^a^	55 (53.9%)	0.047*
Mechanical hyperalgesia	15 (38.5%)	6 (18.8%)	5 (16.1%)	26 (25.5%)	0.059
Dynamic mechanical allodynia	24 (61.5%)^a^	2 (6.3%)^b^	0 (0.0%)^b^	24 (23.5%)	<0.001^*,†^
Cold allodynia	24 (61.5%)^a^	1 (3.1%)^b^	0 (0.0%)^b^	24 (23.5%)	<0.001^*,†^
Hyperpathia/Temporal summation	28 (71.8%)^a^	11 (34.4%)^b^	11(35.5%)^b^	50 (49.0%)	0.001^*,†^

Categorical variables are expressed in absolute numbers and percentages. *P < 0.05, ^†^*P* < 0.0167, pairwise comparisons Bonferroni correction for multiple comparisons; the groups with different letters are statistically different. CPSP, central post-stroke pain; PSP-Non, non-neuropathic post-stroke pain; No-Pain, stroke without pain.

Cold hypoesthesia was more commonly located in the face, upper and lower limbs in CPSP and its spatial profile was significantly different from No-Pain (Fig. 2).

Mechanical hypoalgesia was more frequently detected in CPSP (61.5%) and PSP-Non (62.5%) than in No-Pain (35.5%), *P* = 0.047. Hyperpathia was more frequently detected in CPSP (71.8%, *n* = 28) than in PSP-Non (34.4%, *n* = 11) and in No-Pain (35.5%, *n* = 11), *P* = 0.001 (Table 1).

Spasticity was present in 53.8% CPSP (*n* = 21) versus 25% (*n* = 8) PSP-Non and 9.7% (*n* = 3) *P* < 0.001, and motor weakness was present in 70.3% (*n* = 26) of CPSP, 75% (*n* = 24) PSP-Non and 54.8% (*n* = 17) No-Pain *P* = 0.030, evidencing a tendency of higher impairment in CPSP versus No-Pain (Supplementary Table 9). Active TP were more frequently observed in the PSP-Non group (75%, *n* = 24), whereas they were present in 13.2% *(n* = 5) of the CPSP group, *P* < 0.001 (Supplementary Tables 9 and 10).

### Quantitative sensory testing

**Figure 3:**
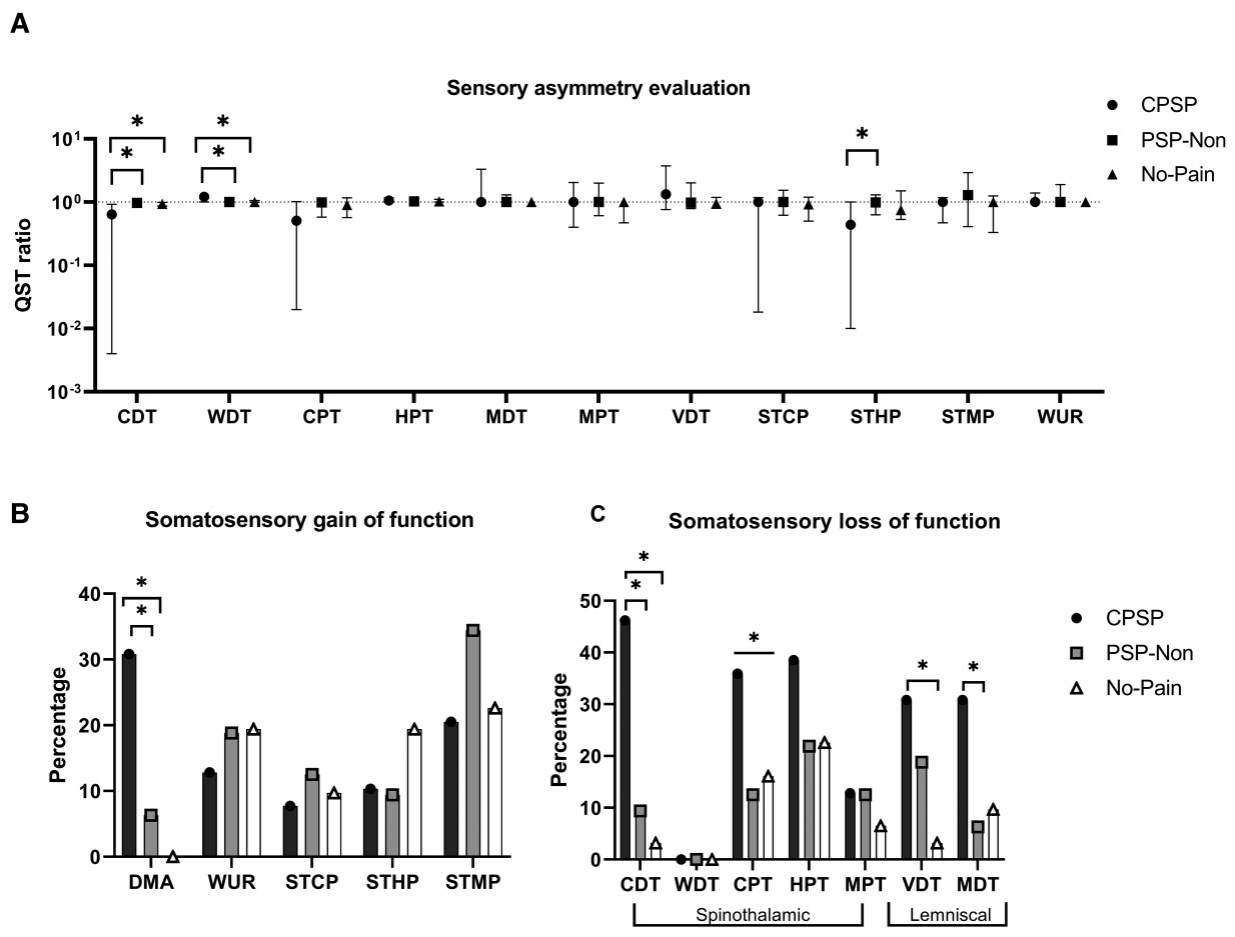
**QST assessment with sensory asymmetry evaluation and somatosensory gain and loss of function.** (**A**) Asymmetry evaluation through the ratio of affected side per unaffected side. The QST ratio is represented as median and interquartile ranges in a log10 scale. Kruskal–Wallis followed by pairwise comparisons were performed using Mann–Whitney for two independent samples with a Bonferroni correction for multiple comparisons. Statistical significance was accepted at the *P* < 0.0167 level.*P < 0.05 for analyses between groups and *P* < 0.0167 for pairwise comparisons. (**B**) Percentage of patients with somatosensory gain of function. *χ*^2^ test and Fisher test were performed followed by pairwise comparisons with Bonferroni correction for multiple comparisons. Statistical significance was accepted at the *P* < 0.0167. (**C**) Percentage of patients with somatosensory loss of function. *P < 0.05 for analyses between groups and *P* < 0.0167 for pairwise comparisons. CPSP, central Post-Stroke Pain; PSP-Non, non-neuropathic post-stroke pain; No-Pain, stroke without pain; CDT, cold detection threshold; WDT, warm detection threshold; MDT, mechanical detection threshold; CPT, cold pain threshold; HPT, heat pain threshold; MPT, mechanical pain threshold; VDT, vibration detection threshold; STCP, suprathreshold cold pain—pain referred according to the Numeric Pain Rating Scale (NRS) after suprathreshold cold pain stimulus; STHP, suprathreshold heat pain—NRS after suprathreshold heat pain stimulus; STMP, NRS after suprathreshold mechanical pain stimulus; WUR, wind up ratio.

- *Side-to-side differences* were measured via the affected area versus mirror area and QST index of asymmetry. CPSP had higher CDT and WDT asymmetry than PSP-Non (*P* < 0.001 and *P* = 0.003) and No-Pain (*P* < 0.001 and *P* = 0.012, respectively), indicating a higher degree of sensory deafferentation. Conversely, mechanical hyperalgesia (STHP) was higher in the PSP-Non group than in CPSP (*P* = 0.007). All QST findings are reported in Tables 2 and 3. None of the other asymmetry scores was significantly different between CPSP and both control groups (Fig. 3).
- *Single-patient classification*: CPSP presented higher percentages of loss of function for spinothalamic tract (STT) (CDT, CPT) and for dorsal column lemniscal-dependent inputs, VDT and MDT (Fig. 3 and Supplementary Table 1^1^). Cold hypoesthesia presented an OR of 12 (95% CI: 3.8–41.6) for neuropathic pain (Supplementary Table 12 for the additional OR of the other QST modalities).
- *Thermal limen assessment*: Patients with CPSP showed values more distant from 1.0 (greater dissociation between cold and warm thermal channels) when compared with the PSP-Non and No-Pain groups (median 0.57 versus 0.95 versus 0.91; *P* < 0.001). Additionally, there was a correlation between this CDT/WDT thermal ratio limen and the presence of neuropathic pain (*ρ* = −0.4, *P* < 0.001) and also with pain intensity (*ρ* = −0.3, *P* < 0.001).

CPM differed between groups (*P* = 0.047), with patients with chronic pain (CPSP and PSP-Non) showing lower values, meaning a defective CPM, but these findings did not persist after multiple comparisons adjustments (Supplementary Table 1^3^). PPT over the reference site (glabella) was similar between groups (2.3 ± 1.24 versus 2.25 ± 0.70 versus 2.58 ± 1.33, *P* = 0.947) though CPSP had lower PPT in all muscles tested compared with PSP-Non and No-Pain (Fig. 2).

**Table 2:** QST evaluation of affected and mirror area according to pain groups

	CPSP, *n* = 39			PSP-Non, *n* = 32			No-Pain, *n* = 31		
Modality	Affected area	Mirror area	Affected ×	Affected area	Mirror area	Affected ×	Affected area	Mirror area	Affected ×
			mirror *P*			mirror *P*			mirror *P*
*Group according to pain classification*									
CDT (°C)	18.8 (0.1–26.5)	29.2 (27.2–29.7)	<0.001*	26.6 (24.3–29.3)	27.7 (25.7–29.9)	0.056	27.7 (25.0–29.0)	29.2 (28.7–30.3)	<0.001*
WDT (°C)	42.8 (35.6–50.0)	34.9 (34.5–36.0)	<0.001*	36.4 (34.9–38.8)	35.6 (34.4–36.7)	0.134	35.2 (34.3–36.5)	34.0 (33.6–34.7)	0.003*
CPT (°C)	1.9 (0.1–14.2)	12.5 (2.8–19.0)	0.003*	9.6 (0.1–21.1)	14.4 (5.2–22.0)	0.150	10.5 (3.7–20.2)	16.3 (8.7–20.7)	0.124
HPT (°C)	50.0 (45.7–50.0)	45.0 (41.1–48.1)	<0.001*	45.9 (41.3 48.1)	44.0 (41.7–46.0)	0.102	47.6 (43.4–49.3)	45.8 (41.1–48.2)	0.023*
MDT (mN)	0.7 (0.2–3.1)	0.2 (0.2–0.7)	0.041*	0.2 (0.2–0.3)	0.2 (0.2–0.2)	0.327	0.2(0.2–0.2)	0.2 (0.2–0.2)	0.207
MPT (mN)	235.2 (81.4–1078.0)	490.3 (81.4–1078.0)	0.777	333.3 (235.2–1078.0)	333.3 (235.2–1078.0)	1.0	490.3 (308.8–1078.0)	1078.0 (333.3–1078.0)	0.084
VDT (mm—64 Hz)	7.0 (2.9–31.0)	4.9 (2.2–8.6)	0.048*	1.9 (1.2–7.5)	1.9 (1.4–4.0)	0.189	1.1 (0.6–1.9)	1.9 (0.7–1.7)	0.658
STCP (NRS)	10.0 (0.1–42.0)	23.5 (6.0–41.5)	0.197	11.0 (0.1–34.5)	15.7 (0.1–41.7)	0.451	15.5 (2.5–50.0)	21.5 (9.5–67.5)	0.070
STHP (NRS)	3.5 (0.1–49.0)	26.0 (13.5–51.5)	0.031*	20.5 (6.5–37.6)	27.0 (6.2–51.6)	0.375	20.0 (8.0–35.0)	18.0 (9.0–55.5)	0.153
STMP (NRS)	2.0 (0.1–28.0)	2.0 (0.1–17.0)	0.851	10.0 (2.2–24.5)	8.5 (1.3–22.2)	0.284	5.0 (0.1–14.0)	5.0 (0.10–15.0)	0.484
WUR	1.0 (1.0–1.4)	1.0 (1.0–1.2)	0.458	1.0 (1.0–1.0)	1.0 (1.0–1.0)	0.330	1.0 (1.0–1.2)	1.0 (1.0–1.0)	0.767
Allodynia 0.1 (0.1–6.0)									

Numerical variables are represented by median and p25 and p75, *P < 0.05. CPSP, central post-stroke pain; PSP-Non, non-neuropathic post-stroke pain; No-Pain, stroke without pain; CDT, cold detection threshold; WDT, warm detection threshold; CPT, cold pain threshold; HPT, heat pain threshold; MDT, mechanical detection threshold; MPT, mechanical pain threshold; VDT, vibration detection threshold; STCP, suprathreshold cold pain stimuli; STHP, suprathreshold heat pain; STMP, suprathreshold mechanical pain stimuli; WUR, wind up ratio (temporal summation). NRS 10° mechanical pain/NRS mechanical pain. NRS, numerical rating scale.

**Table 3:** QST side-to-side comparison between groups

Modality	CPSP *n* = 39	PSP-Non *n* = 32	No-pain *n* = 31	Index comparison between groups	CPSP × PSP-Non	CPSP × No-Pain	PSP-Non × No-Pain
	Side-to-side (index)^Ϭ^	Side-to-side (index)^Ϭ^	Side-to-side (index)^Ϭ^	*P*	*P*	*P*	*P*
CDT	0.6 (0.0–0.9)	1.0 (0.9–1.0)	0.9 (0.9–1.0)	<0.001*	<0.001*,^†^	<0.001*,^†^	0.329
WDT	1.2 (1.0–1.4)	1.0 (1.0–1.1)	1.0 (1.0–1.1)	0.004*	0.003*,^†^	0.012*,^†^	0.379
CPT	0.5 (0.0–1.0)	1.0 (0.6–1.1)	0.9 (0.6–1.2)	0.118			
HPT	1.0 (0.8–1.3)	1.0 (1.1–1.1)	1.0 (1.0–1.1)	0.181			
MDT	1.0 (1.0–3.3)	1.0 (1.0–1.3)	1.0 (1.0–1.0)	0.239			
MPT	1.0 (0.4–2.0)	1.0 (0.6–2.0)	1.0 (0.5–1.0)	0.732			
VDT	1.3 (0.3–120)	1.0 (0.8–2.0)	0.9 (0.8–1.2)	0.195			
STCP	1.0 (0.0–1.2)	1.0 (0.6–1.5)	0.9 (0.5–1.2)	0.332			
STHP	0.4 (0.0–1.0)	1.0 (0.6–1.3)	0.7 (0.5–1.5)	0.012*	0.007*,^†^	0.022*	0.601
STMP	1.0 (0.5–1.2)	1.3 (0.4–2.9)	1.0 (0.3–1.2)	0.421			
WUR	1.0 (1.0–1.4)	1.0 (1.0–1.2)	1.0 (1.0–1.0)	0.608			

Numerical variables are represented by median and p25, and p75. ^Ϭ^Index was calculated according to the formula: affected/mirror. *P < 0.05, †*P* < 0.0167 (pairwise comparisons Bonferroni correction for multiple comparisons). CPSP, central post-stroke pain; PSP-Non, non-neuropathic post-stroke pain; No-Pain, stroke without pain; CDT, cold detection threshold; WDT, warm detection threshold; CPT, cold pain threshold; HPT, heat pain threshold; MDT, mechanical detection threshold; MPT, mechanical pain threshold; VDT, vibration detection threshold; STCP, suprathreshold cold pain stimuli; STHP, suprathreshold heat pain; STMP, suprathreshold mechanical pain stimuli; WUR, wind up ratio (temporal summation): NRS 10° mechanical pain/NRS mechanical pain. NRS, numerical rating scale.

Correlations were found between a number of neuropathic pain symptom clusters and QST modalities in the CPSP group, as follows:

- Paroxysmal pain and CPT (*ρ* = −0.4, *P* = 0.008) and HPT (*ρ* = 0.5, *P* = 0.003);
- Burning pain and MDT (*ρ* = −0.4, *P* < 0.015) and MPT (*ρ* = −0.4, *P* < 0.013).
- Evoked pain and MPT (*ρ* = −0.3, *P* = 0.047) and STMP (*ρ* = −0.3, *P* = 0.032).

There was no correlation between the other two clusters (pressing pain and paresthesia/dysesthesia) and QST modalities (Table 4).

**Table 4:** Correlations between NPSI phenotypes and QST

		*ρ*	*P*
*Correlations NPSI phenotypes and QST modalities*			
Paroxysmal	CPT	0.420	0.008
	HPT	0.460	0.003
Evoked	MPT	−0.320	0.047
	STMP	0.345	0.032
Burning	MDT	−0.387	0.015
	MPT	−0.395	0.013

Correlations were included when *ρ* ≥ 0.3 and *P* < 0.05. CDT, cold detection threshold; WDT, warm detection threshold; CPT, cold pain threshold; HPT, heat pain threshold; MDT, mechanical detection threshold; MPT, mechanical pain threshold; VDT, vibration detection threshold; STCP, suprathreshold cold pain stimuli; STHP, suprathreshold heat pain; STMP, suprathreshold mechanical pain stimuli. For asymmetry index calculation: a ratio (values from tested area/values from the mirror area) for CDT, WDT, MDT, MPT, VDT, STCP, STHP, STMP. For CPT and HPT evaluation, the difference between values of tested and mirror area (tested area − minor area). There was no correlation between pressing and parestheasia phenotypes and QST.

### Multivariate analyses

We performed a binomial logistic regression including variables found to be different between groups from pain descriptors (NPSI), from clinical bedside examination (allodynia), and from QST (CDT in painful side versus mirror area < 41%) and the likelihood these patients would have been classified as being from the CPSP group. The model was statistically significant [*χ*²(3) = 85.1, *P* < 0.001] and explained 77% (Nagelkerke R2) of the variance in CPSP. Of the variables, all were statistically significant: cold hypoesthesia OR = 8.1, 95% CI = 1.6–42.5; NPSI OR = 1.1, 95% CI = 1.0–1.1; NRS allodynia OR = 2.1, 95% CI = 1.3–3.7.

Employing dichotomous variables on the model NPSI (≥20) and NRS allodynia (≥2), the model was statistically significant [*χ*²(3) = 51.3, *P* < 0.001] and explained 69% (Nagelkerke *R*^2^) of the variance in CPSP. Of the variables, all were statistically significant: cold hypoesthesia OR = 7.4, 95% CI 1.3–43.6; NPSI OR = 11.3, 95% CI 2.4–53.4; allodynia OR = 36.9, 95% CI 3.7–370.6.

## Discussion

We have reported on symptom profile correlations with sensory characteristics of CPSP patients compared with matched pain-free and PSP without neuropathic pain groups. This was an original approach to dissect what in CPSP is specific to this condition relative to other post-stroke chronic pain syndromes or stroke in general.

CPSP patients reported more intense pain, higher pain sensory and affective sub-scores, as well as a trend towards more functional impairment compared with non-neuropathic PSP patients. The quality of pain was also different between groups, with CPSP being more frequently continuous, burning, tingling and evoked by cold stimuli, compared with non-neuropathic PSP, which was, in turn, more commonly deeply located, intermittent, as pressure and never reported as tingling or as electric shocks.

At bedside examination, cold/mechanical dynamic allodynia occurred mainly in CPSP and was present in the large majority of these patients. Also, hyperpathia was one of the most frequent signs found in the CPSP group, present in more than 70% of CPSP patients. While dysesthesia, allodynia or hyperalgesia have been reported to predict CPSP,^98^ hyperpathia remains a relatively underexplored sign that may also be a useful predictor of CPSP, and was previously reported to be prevalent in this condition.^34,95,100^

Using standardized manikin-based assessment, we found that CPSP was distributed over more extensive and also spatially diverse body areas, such as the face, arms, legs or hemi-body, which contrasted with PSP-Non where the pain was present more frequently axially: in the neck, shoulders and knees.^18,38,82,84,95,101–104^ Also, CPSP had more thermal detection deficits in the painful area compared with the two control groups under QST. This is one of the main findings of this study since these differences were present not only in side-to-side asymmetry within each patient, but also in comparison with both control groups. We found that CPSP has a significantly disproportionately higher asymmetry in WDT and CDT compared with controls and these differences, explored by the sensory limen, correlated with the presence of neuropathic pain. This finding is original and is in line with several studies on experimental thermal allodynia triggered by the thermal grill illusion of pain showing that higher differences between non-painful cold and warm are responsible for more intense and more robust thermal heat allodynia as triggered by the technique.^105^ Similarly, thermal deficit asymmetry was the only variable discriminating between pain and pain-free syringomyelia patients.^106^ These findings are also in accordance with the report that patients with Wallenberg’s syndrome central pain was less frequent when thermal abnormalities tended towards symmetry.^38^ Indeed, it has been proposed that more rostral sites of CNS lesions would affect sensory modalities in a more disproportionate manner compared with spinal lesions so that more cranial lesions would dissociate warm/cold and mechanical thresholds more markedly compared with spinal lesions.^13^

This was the first description of neuropathic symptom cluster profiles and their relationships with QST in CPSP compared with PSP pain of non-neuropathic nature. Four out of the five original neuropathic pain symptom clusters were more common in CPSP, except for deep spontaneous pain. In CPSP, burning (superficial) spontaneous pain was the symptom cluster with the highest scores, followed by paresthesia/dysesthesia and evoked pain.^18,31,38,82,95,96,100,107^ This contrasts with peripheral neuropathic pain, where cold-induced pain is less frequently reported.^40,108–111^ We found a positive correlation between abnormal thermal pain thresholds and paroxysmal pain, while altered MPTs correlated with burning pain/evoked pain scores. Similar correlations between paroxysmal pain intensity and thermal sensitivity were reported in peripheral neuropathic pain studies.^112–114^ It has also been reported that patients with syringomyelia having exclusively spontaneous pain (which included paroxysmal pain) had more asymmetrical and more severe thermal deficits, while patients with allodynia had less affected thermal deficits.^115^ Contrarily, a large body of evidence from human neurophysiology studies assessing thinly myelinated (e.g. laser-evoked potentials) and large-myelinated [e.g. somatosensory evoked potentials (SEPs)] have suggested that continuous ongoing pain would be related to injuries affecting small fibres, while paroxysmal pain would be related to lesions to myelinated large fibres.^116^ However, even in these reports, these distinctions are not unequivocal: in patients with multiple sclerosis, about a third of those with pain due to Lhermitte sign’s (shock-like triggered though the neck-dorsum by neck flexion) had normal SEPs, while SEPs were abnormal in about a third of those presenting with ongoing extremity pain.^117^ Importantly, most hypothesis linking myelinated fibre lesions leading to abnormal discharges and paroxysmal pain relies on an otherwise normal second/third order wide-dynamic range neurons that would receive high-frequency discharges conveyed by injured myelinated fibres (from peripheral nerves^118^ or from the dorsal column lemniscal pathways^117,119^) and would then divert them into nociceptive pathways, where discharges would eventually be perceived as painful. In central neuropathic pain, 2nd or 3rd order sensory neurons are frequently included within the lesion area, thus potentially altering the central processing of thermo-nociceptive inputs. It must also be kept in mind that correlations found here and elsewhere do not imply causality, and may be due to an undetermined mediating cofactor between paroxysms and thermal thresholds abnormalities such as the severity of lesion.

Previous studies on central neuropathic pain have reported that patients had altered spinothalamic-dependent abnormalities, while lemniscal pathways could be either intact or affected.^18,34,82,84,120,121^ This has led to the imbalance theory,^122^ postulating that CPSP would occur due to residual lemniscal inputs arriving in the absence of STT information in higher-order neurons. Our results are in line with such a view, since QST-based thermal cold hypoesthesia carried the highest OR for CPSP (=12.0), and that CPSP patients had more widespread sensory abnormalities. However, classic QST batteries offer a relatively limited assessment of lemniscal function and one cannot refute that concomitant lemniscal abnormalities were not present in our samples.

Most clinically relevant results came from the logistic regression. The association of pain NPSI score, presence of allodynia on bedside examination and CDT abnormalities on QST (CDT from painful side/mirror side < 41%^87^), explained 77% of the occurrence of neuropathic pain. Interestingly, this model comprises the basic steps in the clinical diagnosis of neuropathic pain: use of pain descriptors, presence of abnormal sensory gain on bedside examination and the determination of STT-related deficits (CDT). This may potentially be useful information in the distinction between neuropathic from non-neuropathic PSP and may help better design interventional trials in the future.

One important finding, with potential impact on CPSP definitions, and how to differentiate it from its mimics, was that a third of non-neuropathic PSP patients had their pain located within the sensory deficit area. These patients did not fulfill the criteria for neuropathic pain and had other clear causes of non-neuropathic pain such as headaches and musculoskeletal pain. This finding has been previously reported for spinal cord pain,^79^ but not yet in CPSP. This information has clinical relevance and calls attention to the necessity to have pain descriptors included in neuropathic pain definitions, as well as to the requirement to proactively search for sources of nociceptive pain within the deafferented area in a patient with clear neuropathy.^5^

Similar to others^18,31,34,36–39,84–86,100^ (Table 5), we performed QST in the area with more intense pain, and this region may not necessarily be the body area with more prominent sensory abnormalities. In fact, we have shown that the sensory deficit area is not only wider, but qualitatively different between groups, and sensory assessments based on the area of maximal pain may miss areas with maximal sensory denervation or with non-painful sensory gain of function. This also highlights the challenge related to the choice of the control area in central pain studies. In these instances, since the painful area may vary significantly in body location across individuals from the same experimental group, control areas cannot be compared with healthy volunteers-based normative data and are, instead, based on the same rationale used during the neurological examination, comparing dermatomes above and below the sensory level in spinal cord injury^79^ or syringomyelia,^106,115^ or the mirror area in cases of stroke.^18,31,34,36–39,84–86^ Another challenge is related to the inclusion and assessment of patients with stroke-related acquired language dysfunction. Here, patients with cognitive impairment were excluded in order to perform a detailed assessment of pain descriptors and sensory profiles. This is, however, a limitation of the external validity of our results since only language-spared patients were assessed and our findings may not apply to those with different degrees of aphasia.

**Table 5:** Quantitative sensory test studies for central post-stroke pain investigation

Study	Patient sample	Control group	Methods	Findings
Boivie *et al.*^34^	27 CPSP (eight brainstem lesion, nine thalamic, six suprathalamic and four undetermined)		Area: feet, hand and face versus contralateral side Abnormal: thresholds at least twice as high as the control side.	All had abnormal temperature and pain sensibility: Hypoalgesia: 37% Mechanical hypoesthesia: 52% Abnormal vibration sensibility: 41% Hyperpathia: 88% Hyperalgesia: 60% Dysesthesia: 85% Allodynia: 23%
Leijon and Bowsher^39^	36 CPSP	13 stroke with a sensory deficit and without CPSP	Area: side with symptoms and mirror Method of limits Descriptive analysis	Cold, warm and cold pain thresholds abnormalities: 89 versus 50% Heat pain thresholds—normal in all subjects. Abnormal tactile sensation: 86 versus 38%, Abnormal pinprick sensation: 86 versus 54%,
				Allodynia: 57% (28% to touch, 42% to cold) versus 0%. All CPSP had cold, warm, or pinprick abnormalities.
Vestergaard *et al.*^84^	11 CPSP All had a supratentorial lesion (five thalamic, six solely extrathalamic, seven also brainstem)		Area: worst pain area (all in the thenar eminence) and mirror area Methods of limits Statistical comparison between pain area and mirror	Increased threshold of thermal (cold 91%, warm 100%) Abnormal sensibility to pain 36% Abnormal sensibility to touch 27% Allodynia: 72.7% (cold 56%, touch 54%)
Bowsher^31^	74 central 61 CPSP		Measures at four sites: The greatest pain area and its mirror, and the least pain area and its mirror Methods of limits Statistical analysis between greatest pain versus mirror and least pain versus mirror Greatest pain versus least pain (*P* < 0.05)	Greatest versus least pain: Significant for pinprick, warm and cold. All modalities were significant for greatest versus mirror and least versus mirror. 72% allodynia (52% tactile, 19.5% thermal, 22% movement)
MacGowan *et al*.^38^	9 CPSP with Wallenberg syndrome	10 Wallenberg syndrome without CPSP	Standard areas tested bilaterally Method of limits/forced-choice Comparison to healthy controls (classified as elevated or not)	CPSP-thresholds from the cheek contralateral to the lesion were normal in eight of nine cases with CPSP and abnormal in all 10 cases without CPSP. CPSP allodynia—mechanical (50%) cold (75%)
				CPSP allodynia—mechanical (50%) cold (75%)
Bowsher *et al.*^18^	32 CPSP VPL 21 Brainstem 11	20 stroke patients with a sensory deficit and without CPSP	Side with symptoms and mirror Methods of limits The difference between affected side and mirror compared between CPSP and control	CPSP and control had differences comparing maximally affected and mirror areas for warm, cold, pinprick and heat pain. VPL versus control: differences for pinprick and cold detection. Brainstem versus control: differences for pinprick, cold and warm and hot pain. VPL versus brainstem: differences only for warm detection.
Fitzek *et al.*^85^	Eight patients with Wallenberg syndrome and CPSP	Four patients with Wallenberg syndrome without CPSP	Both sides of the face (upper cheek). Method of limits Statistical comparison between affected and mirror area	Cold and warm detection, cold and heat pain and touch thresholds in the ipsilateral face versus mirror were significantly different in all patients with facial pain but not in patients without pain.
Greenspan *et al.*^37^	13 CPSP		Affected and mirror area Method of limits Abnormal threshold: the value of the mean ± 2SD outside the normative range Included absolute values and difference between the affected and unaffected side	Cold hypoesthesia: 84.6% Warm hypoesthesia:92.3% Cold hypoalgesia: 46.1% Warm hypoalgesia: 7.6% Tactile hypoesthesia: 38.5% Cold allodynia: 23% Brushing allodynia: 53.8% More tactile allodynia in individuals with normal tactile detection.
Bowsher^86^	64 CPSP		Means of somatosensory perception threshold differences (affected − mirror)	About half of patients with CPSP had allodynia Pure cold allodynia versus cold plus mechanical allodynia: affected–unaffected cold threshold difference greater in the latter, but not significant (*P* = 0.06)
Kalita *et al.*^100^	23 CPSP		QST, SPECT and MRI	Reduced pain threshold: 43.5% Increased pain threshold: 56.5% About half of CPSP had allodynia, temporal summation, or punctate hyperalgesia: Findings were similar in patients with thalami and extra thalamic lesions. SPECT and MRI findings were not different in CPSP patients with and without allodynia.
Krause *et al.*^36^	25 CPSP	25 sensory stroke without pain	Area of painful sensation and mirror confined to either the face, hand or foot. *Z*-score	CPSP: alterations of thermal and mechanical thresholds on the affected side. Higher values for paradoxical heat sensation and dynamic mechanical allodynia and elevated cold detection threshold. Sensory stroke: similar albeit less pronounced changes in thermal and mechanical thresholds. Both groups: considerable QST changes on the unaffected side.

CPSP, Central post-stroke pain; VPL, ventroposterior thalamic nucleus.

It has been proposed that, compared with healthy volunteers-based normative data,^36^ stroke patients may present subtle sensory abnormalities even in the normal body side. It remains unknown whether the origin of these ipsilateral changes is related to concomitant diseases associated with stroke (e.g. diabetic polyneuropathy), to bias related to slower reaction time in stroke patients, or to maladaptive plasticity after stroke. The fact is that these reports highlight the need to have control groups with pain and stroke in order to account for these abnormalities ipsilateral to the stroke side.

In summary, we reported, in a double-controlled study, that CPSP was associated with thermal detection deficits, allodynia, hyperpathia, on bedside assessment, and several of the symptom clusters of CPSP were correlated to discrete QST parameters. Also, we showed that a combination of neuropathic pain symptoms, the presence of cold detection deficits and allodynia explain a large proportion of the occurrence of CPSP. These findings will have diagnostic utility and may help better design personalized treatments based clinical and QST findings for CPSP in the near future.

## Supplementary Material

fcac090_Supplementary_Data

## Abbreviations

CDT =: cold detection threshold
CPM =: conditioned pain modulation
CPSP =: central post-stroke pain
CPT =: cold pain threshold
DN-4 =: Douleur Neuropathique Questionnaire-4
HPT =: heat pain threshold
IASP =: International Association for the Study of Pain
m-AS =: modified-Ashworth spasticity scale
MDT =: mechanical detection threshold
MPT =: mechanical pain threshold
MRC =: Medical Research Council
mRS =: modified Rankin scale
NeuPSIG/IASP =: IASP Special Interest Group on Neuropathic Pain
No-Pain =: post-stroke patients without chronic pain
NPSI =: Neuropathic Pain Symptoms Inventory
NRS =: numeric rating scale
PDQ =: Pain Disability Questionnaire
PPT =: pressure pain threshold
PSP-Non =: non-neuropathic post-stroke pain
QST =: quantitative sensory testing
QuickDash =: shortened disabilities of the arm, shoulder and hand questionnaire
SEP =: somatosensory evoked potential
SF-12 =: The Short Form 12-Health Status Questionnaire
SF-MPQ =: Short-form McGill Pain Questionnaire
STCP =: numerical pain rating scale for suprathreshold cold stimuli
STHP =: suprathreshold heat pain
STMP =: numerical pain rating scale suprathreshold mechanical stimuli
STT =: spinothalamic tract
TP =: myofascial trigger points
VDT =: vibration detection threshold
WDT =: warm detection threshold
WUR =: wind up ratio
