# Supplementary material for: Dissecting central post-stroke pain: a controlled symptom-psychophysical characterization
Source: Brain Commun. 2022 Apr 5;4(3):fcac090. doi: 10.1093/braincomms/fcac090 (PMC9070496; doi:10.1093/braincomms/fcac090)
Supplement: fcac090_Supplementary_Data [file fcac090_supplementary_data.docx]

Supplementary Figure 1: Proportion of patients with Post-Stroke non-neuropathic pain in an area with sensory abnormality.


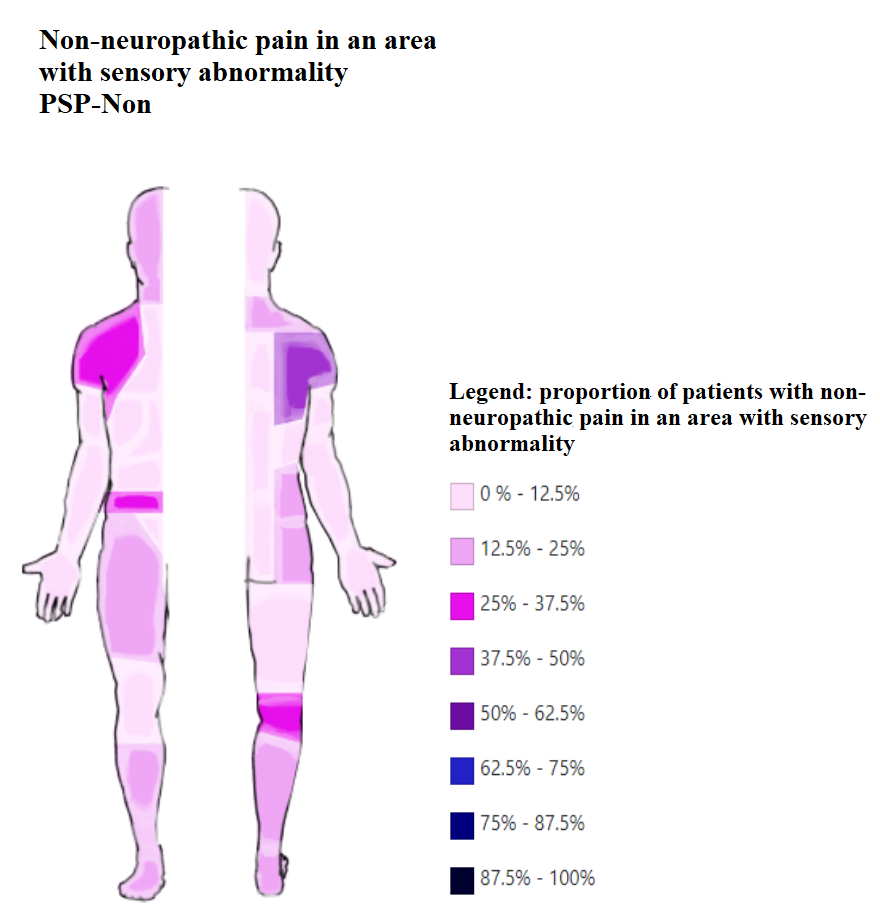


N=12 PSP-Non: Post-Stroke non-neuropathic pain

Supplementary Figure 2: NPSI total score ROC curve analysis


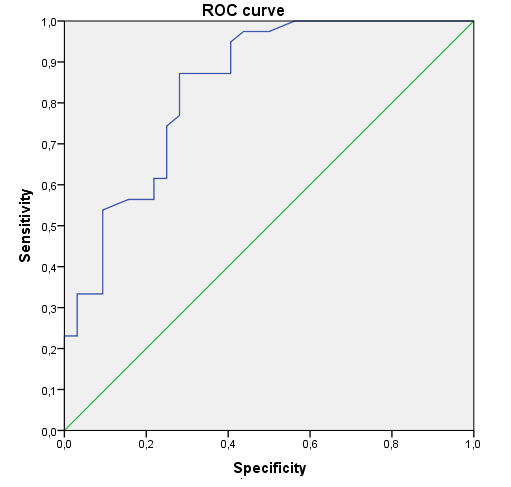


Total Neuropathic pain symptom inventory (NPSI) cut-off for detecting neuropathic pain. Diagonal segments are produced by ties

Supplementary Figure 3: Dynamic mechanical allodynia and cold allodynia cut-off point for neuropathic pain

*
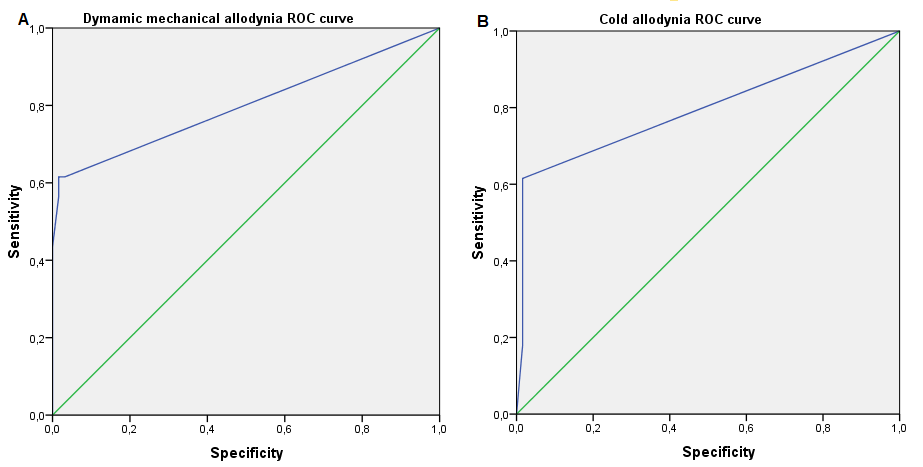
*

A: Numerical Rating Pain Scale reported for Dynamic mechanical allodynia ROC curve analysis for Neuropathic pain. B: Numerical Rating Pain Scale reported Cold allodynia ROC curve analysis for Neuropathic pain. Diagonal segments are produced by ties

Supplementary Figure 4- The Neuropathic Pain Symptoms Inventory (NPSI) presented in five clusters


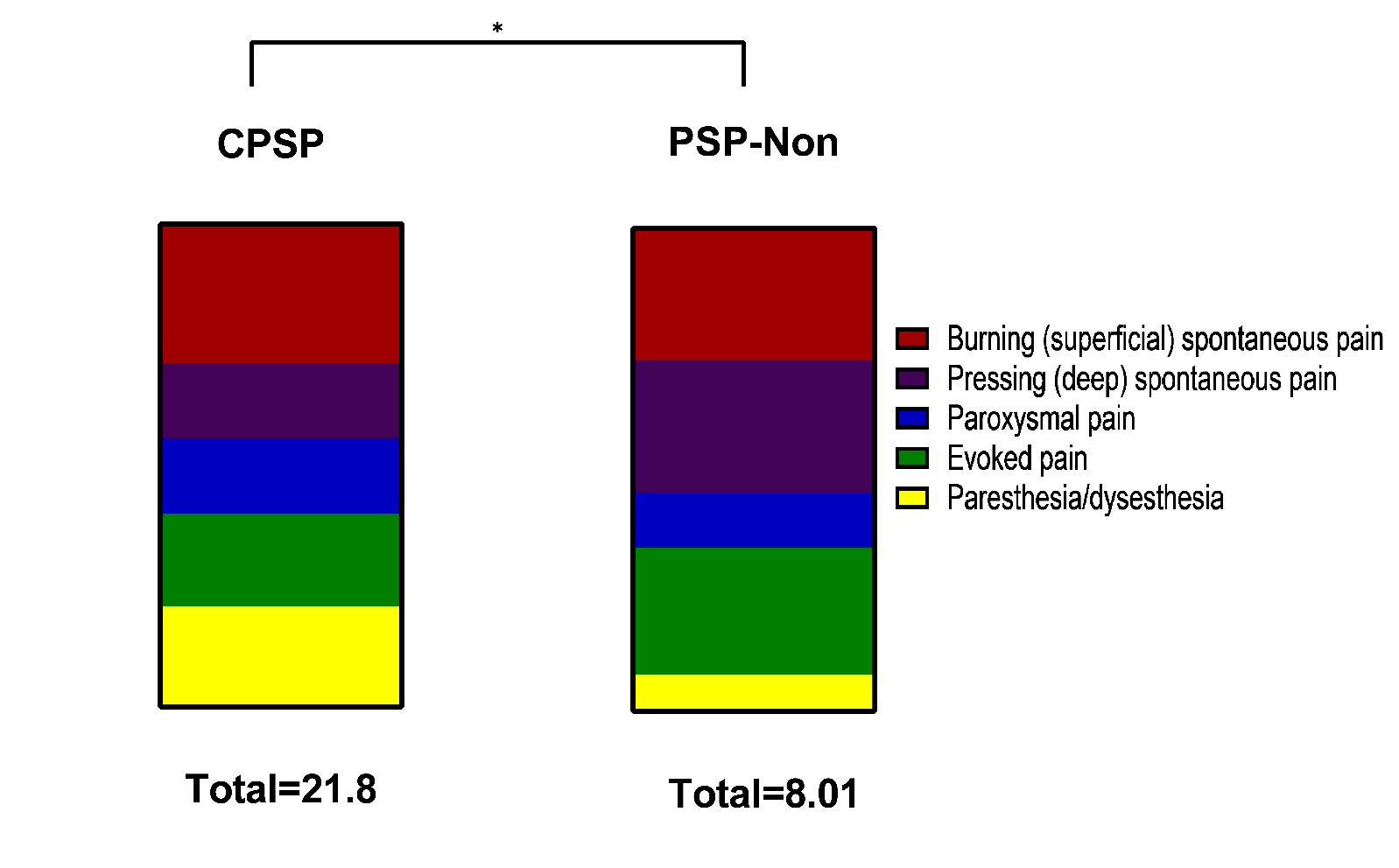


* p<0.005, Mann Whitney test for two independent samples. NPSI score varied from 0 to 50 and is represented as mean. Central Post-Stroke Pain. PSP-Non: Non-neuropathic post-stroke pain. Post-Stroke Pain.

Supplementary Table 1- Sociodemographic characteristics and comparative analysis between stroke patients grouped according to pain syndrome**.**

|  | Group according to pain classification | | | |  |  |  |  |
| --- | --- | --- | --- | --- | --- | --- | --- | --- |
|  | CPSP  n =39 | PSP-Non  n =32 | NO-Pain  n =31 | Total  n =102 | P between groups | CPSP x PSP-Non | CPSP x No-Pain | PSP-Non x  No-Pain |
| Age (years) | 59.2(11.2) | 61.9 (11.1) | 57.1(13.3) | 59.4(11.9) | 0.281 |  |  |  |
| Sex (Male) | 23 (59.0%) | 19 (59.4%) | 24 (77.4%) | 66 (64.7%) | 0.207 |  |  |  |
| Educational level | | | | | |  |  |  |
| Low^a^ | 14 (35.9%) | 19 (59.4%) | 15 (48.4%) | 48 (47.1%) | 0.055 |  |  |  |
| Medium^b^ | 19 (48.7%) | 10 (31.3%) | 7 (22.6%) | 36 (35.3%) |  |  |  |  |
| High^c^ | 6 (15.4%) | 3 (9.4%) | 9 (29.0%) | 18 (17.6%) |  |  |  |  |
| Working | 2 (5.1%) | 6 (18.8%) | 11 (35.5%) | 19 (18.6%) | 0.005* | 0.071 | 0.001^†^ | 0.135 |

Categorical variables are expressed in absolute numbers and percentages. Numerical variables are represented by mean and standard deviation. ^*^p<0.05, ^†^ p<0.0167 (pairwise comparisons Bonferroni correction for multiple comparisons).^a^Low: middle, elementary school or no education. ^b^Medium: high school. ^c^High: bachelor's degree or higher. CPSP: Central Post-Stroke Pain. PSP-Non: Non-neuropathic post-stroke pain. Post-Stroke Pain. No-Pain: Stroke without pain.

Supplementary Table 2-Medical comorbidity and Body Mass Index characteristics and comparative analysis between stroke patients grouped according to pain syndrome.

|  | Group according to pain classification | | | | | |  | |  | |  |  |
| --- | --- | --- | --- | --- | --- | --- | --- | --- | --- | --- | --- | --- |
|  | CPSP  n=39 | PSP-Non  n =32 | NO-Pain  n=31 | | Total  n =102 | | P between  groups | | CPSP x PSP-Non | | CPSP x No-Pain | PSP-Non x No-Pain |
| Medical history | | | | | | | | | | | |  |
| Diabetes | 10(25.6%) | 13(40.6%) | 8 (25.8%) | 31(30.4%) | | 0.315 | |  | |  | |  |
| Hypertension | 33(84.6%) | 28(87.5%) | 24(77.4%) | 85(83.3%) | | 0.542 | |  | |  | |  |
| Heart disease | 8 (20.5%) | 16(50.0%) | 14(45.2%) | 38(37.3%) | | 0.021^*^ | | 0.009^†^ | | 0.027 | | 0.701 |
| CKD | 1 (2.6%) | 7 (21.9%) | 2 (6.5%) | 10 (9.8%) | | 0.025 ^*^ | | 0.019 | | 0.580 | | 0.148 |
| Depression | 10(25.6%) | 6 (18.8%) | 4 (12.9%) | 20(19.6%) | | 0.407 | |  | |  | |  |
| Currently smoking: | 7 (17.9%) | 4 (12.5%) | 3 (9.7%) | 14(13.7%) | | 0.674 | |  | |  | |  |
| Body Mass Index(Kg/m²) | 28.0 (4.6) | 26.1 (3.7) | 27.0(4.4) | 27.1(4.3) | | 0.136 | |  | |  | |  |

Categorical variables are expressed in absolute numbers and percentages. Numerical variables are represented by mean and standard deviation ^*^p<0.05, ^†^ p<0.0167 (pairwise comparisons Bonferroni correction for multiple comparisons).CPSP: Central Post-Stroke Pain. PSP-Non: Non-neuropathic post-stroke pain. Post-Stroke Pain. NO-Pain: Stroke without pain. CKD: Chronic kidney disease.

Supplementary Table 3: Stroke characteristics regarding the type of event, location and symptomatic side, and comparative analysis between stroke patients, grouped according to the pain syndrome.

|  | Group according to pain classification | | | | |
| --- | --- | --- | --- | --- | --- |
|  | CPSP | PSP-Non | No-Pain | Total | p |
| Time elapsed after stroke | n= 38  55.1( 58.0) | n =32  51.0 (38.9) | n= 31  35.2(24.0) | n =101  47.7 (44.3) | 0.268 |
| Type of event | N= 38 | N =32 | N =31 | N =101 |  |
| Hemorrhagic | 8 (21.1%) | 2 (6.3%) | 4 (12.9%) | 14 (13.9%) | 0.179 |
| Ischemic | 30 (78.9%) | 30 (93.8%) | 27 (87.1%) | 87 (86.1%) |  |
| Stroke side | N=39 | N=32 | N=31 | N=102 |  |
| Right | 22 (56.4%) | 14 (43.8%) | 10 (32.3%) | 46 (45.1%) |  |
| Left | 11 (28.2%) | 12 (37.5%) | 17 (54.8%) | 40 (39.2%) | 0.229 |
| Bilateral | 6 (15.4%) | 6 (18.8%) | 4 (12.9%) | 16 (15.7%) |  |
| Symptomatic side |  |  |  |  |  |
| Right | 14 (35.9%) | 13 (40.6%) | 14 (45.2%) | 41 (40.2%) | 0.064 |
| Left | 22 (56.4%) | 14 (43.8%) | 8 (25.8%) | 44 (43.1%) |  |
|  |  |  |  |  |  |
| Stroke location |  |  |  |  |  |
| Cortical | 11 (28.2%) | 13 (40.6%) | 15 (48.4%) | 39 (38.2%) | 0.178 |
| Subcortical | 20 (51.3%) | 11 (34.4%) | 7 (22.6%) | 38 (37.3%) |  |
| Brainstem and cerebellum | 8 (20.5%) | 8 (25.0%) | 9 (29.0%) | 25 (24.5%) |  |
| More than one lesion | 8 (20.5%) | 9 (28.1%) | 4 (12.9%) | 21 (20.6%) | 0.342 |

Categorical variables are expressed in absolute numbers and percentages. Numerical variables are represented by mean and standard deviation.^Ɨ^ CPSP: Central Post-Stroke Pain. PSP-Non: Non-neuropathic post-stroke pain. Post-Stroke Pain. NO-Pain: Stroke without pain

Supplementary Table 4: Results of The Brief Pain Inventory scores

| **The Brief Pain Inventory** | | | | |
| --- | --- | --- | --- | --- |
|  | Group according to pain classification | | | |
|  | CPSP  n= 39 | PSP-Non  n= 32 | Total  n=71 | P effects between groups |
| Severity items (pain) | | | |  |
| Least (0-10) | 5.1 (2.2) | 2.9 (1.8) | 4.1 (2.3) | <0.001^*^ |
| Average (0-10) | 6.5 (1.6) | 4.8 (2.6) | 5.7 (2.2) | 0.003^*^ |
| Now (0-10) | 6.6 (1.8) | 3.2 (3.1) | 5.1 (3.0) | <0.001^*^ |
| Worst (0-10) | 7.5 (1.7) | 6.5 (3.3) | 7.1 (2.6) | 0.493 |
| Interference items |  |  |  |  |
| General activity (0-10) | 5.4 (3.5) | 3.8 (3.4) | 4.7 (3.6) | 0.055 |
| Mood (0-10) | 4.9 (3.9) | 3.8 (3.7) | 4.4 (3.8) | 0.265 |
| Walking ability (0-10) | 4.9 (4.0) | 4.4 (3.8) | 4.7 (3.9) | 0.531 |
| Normal work (0-10) | 3.9 (3.8) | 4.2 (3.2) | 4.0 (3.5) | 0.771 |
| Relationships with others (0-10) | 3.9 (4.2) | 2.5 (3.3) | 3.3 (3.9) | 0.185 |
| Sleep (0-10) | 4.1 (4.3) | 3.7 (3.9) | 3.9 (4.1) | 0.708 |
| Enjoyment of life (0-10) | 5.3 (4.1) | 4.1 (3.8) | 4.72 (4.0) | 0.225 |
| Percentage of relief provided by pain treatment (0-100%) | 41.3 (25.8) | 52.9 (38.1) | 46.1 (31.8) | 0.141 |

Categorical variables are expressed in absolute numbers and percentages. Numerical variables are represented by mean and standard deviation. *p<0.05. CPSP: Central Post-Stroke Pain. PSP-Non: Non-neuropathic post-stroke pain. Post-Stroke Pain.

Supplementary Table 5: Short-Form of the McGill pain questionnaire.

| Short-form McGill | | | | |
| --- | --- | --- | --- | --- |
|  | Group according to pain classification | | |  |
|  | CPSP  n= 39 | PSP-Non  n= 32 | Total  n=71 | P |
| Total score (0-15) | 11.2 (2.8) | 7.8 (3.3) | 9.6 (3.5) | <0.001 ^*^ |
| Sensory (0-8) | 5.7 (1.7) | 3.5 (2.0) | 4.7 (2.1) | <0.001 ^*^ |
| Affective (0-5) | 3.9 (1.4) | 2.9 (1.4) | 3.4 (1.4) | 0.003 ^*^ |
| Evaluative (0-2) | 1.6 (0.5) | 1.4 (0.6) | 1.5 (0.6) | 0.075 |

Numerical variables are represented by mean and standard deviation. *p<0.05

Supplementary Table 6: Results of the neuropathic pain symptoms inventory.

|  | CPSP n=39 | PSP-Non n=32 | Total n=71 | P |
| --- | --- | --- | --- | --- |
| Pain Descriptor (items) Number and Percentage of patients who reported a score > 0 | | | | |
| Burning | 32 (82.1%) | 10 (31.3%) | 42 (59.2%) | <0.001^*^ |
| Squeezing | 15 (38.5%) | 8 (25.0%) | 23 (32.4%) | 0.228 |
| Pressure | 20 (51.3%) | 11 (34.4%) | 31 (43.7%) | 0.153 |
| Electric shocks | 23 (59.0%) | 0 (0.0%) | 23 (32.4%) | <0.001^*ß^ |
| Stabbing | 17 (43.6%) | 7 (21.%) | 24 (33.8%) | 0.054 |
| Evoked by brushing | 18 (46.2%) | 4 (12.5%) | 22 (31.0%) | 0.002 ^*^ |
| Evoked by pressure | 24 (61.5%) | 21 (65.6%) | 45 (63.4%) | 0.722 |
| Evoked by cold stimulus | 25 (64.1%) | 2 (6.3%) | 27 (38.0%) | <0.001^*ß^ |
| Pins and needles | 22 (56.4%) | 5 (15.6%) | 27 (38.0%) | <0.001^*ß^ |
| Tingling | 26 (66.7%) | 0 (0.0%) | 26 (36.6%) | <0.001^*ß^ |
| Spontaneous pain during the last 24h | | | | 0.003^*^ |
| Permanently | 26 (66.7%) | 9 (28.1%) | 35 (49.3%) |  |
| Between 8 and 12 h | 3 (7.7%) | 6 (18.8%) | 9 (12.7%) |  |
| Between 4 and 7 h | 5 (12.8%) | 9 (28.1%) | 14 (19.7%) |  |
| Between 1 and 3h | 2 (5.1%) | 1 (3.1%) | 3 (4.2%) |  |
| Less than 1 h | 3 (7.7%) | 7 (21.9%) | 10 (14.1%) |  |
| Pain attacks during the last 24h |  |  |  | <0.001^*^ |
| More than 20 | 9 (23.1%) | 0 (0.0%) | 9 (12.7%) |  |
| Between 11 and 20 | 3 (7.7%) | 0 (0.0%) | 3 (4.2%) |  |
| Between 6 and 10 | 5 (12.8%) | 2 (6.3%) | 7 (9.9%) |  |
| Between 1 and 5 | 10 (25.6%) | 3 (9.4%) | 13 (18.3%) |  |
| No pain attack | 12 (30.8%) | 27 (84.4%) | 39 (54.9%) |  |
| NPSI scores (0-10) |  |  |  |  |
| Burning | 6.28 (3.58) | 2.22 (3.54) | 4.45 (4.08) | <0.001^*^ |
| Squeezing | 2.87 (3.97) | 1.97 (3.49) | 2.46 (3.76) | 0.344 |
| Pressure | 3.87 (4.17) | 2.59 (3.82) | 3.30 (4.03) | 0.162 |
| Electric shocks | 4.08 (3.76) | 0.00 (0.00) | 2.24 (3.44) | <0.001^*^ |
| Stabbing | 2.72 (3.47) | 2.06 (3.741) | 2.42 (3.58) | 0.345 |
| Evoked by brushing | 3.28 (3.80) | 1.00 (2.82) | 2.25 (3.56) | 0.005^*^ |
| Evoked by pressure | 4.26 (3.77) | 5.03 (4.08) | 4.61 (3.90) | 0.351 |
| Evoked by cold stimulus | 5.05 (4.22) | 0.47 (1.88) | 2.99 (4.062) | <0.001^*^ |

Categorical variables are expressed in absolute numbers and percentages. Numerical variables are represented by mean and standard deviation. *p<0.05

Supplementary Table 6: continuation Results of the neuropathic pain symptoms inventory

| NPSI scores (0-10) | CPSP n=39 | PSP-Non n=32 | Total n=71 | P |
| --- | --- | --- | --- | --- |
| Pins and needles | 3.8 (3.8) | 1.4 (3.0) | 2.7 (3.7) | 0.003^*^ |
| Tingling | 5.1 (4.1) | 0.00 (0.00) | 2.8 (4.0) | <0.001^*^ |
| NPSI total intensity score (0-100) | 41.3 (20.7) | 16.1 (17.1) | 29.9 (22.9) | <0.001^*^ |
| NPSI five clusters (0-10) |  |  |  |  |
| Burning (superficial) spontaneous pain | 6.3 (3.6) | 2.2 (3.5) | 4.4 (4.1) | <0.001^*^ |
| Pressing (deep) spontaneous pain | 3.4 (3.4) | 2.2 (2.9) | 2.8 (3.2) | 0.145^β^ |
| Paroxysmal pain | 3.4 (3.0) | 0.9 (1.8) | 2.7 (2.8) | <0.001^*^ |
| Evoked pain | 4.2 (2.9) | 2.1 (2.0) | 3.3 (2.7) | 0.002^*^ |
| Paresthesia/dysesthesia | 4.5 (3.1) | 0.6 (1.5) | 2.7 (3.2) | <0.001^*^ |
| Sum of subscores score (0-50) | 21.7 (10.4) | 8.1 (8.8) | 15.6 (11.8) | <0.001^*^ |
| NPSI three clusters (Bouhassira D. *et al.* 2021*)* | | | | |
| Deep pain | 14 (35.9%) | 26 (81.3%) | 40 (56.3%) | <0.001 ^*^ |
| Provoked pain | 15 (38.5%) | 6(18.8%) | 21(29.6%) |  |
| Pinpointed pain | 10 (25.6%) | 0 | 10(14.1%) |  |

Categorical variables are expressed in absolute numbers and percentages. Numerical variables are represented by mean and standard deviation. *p<0.05

Supplementary Table 7: The Hospital Anxiety and Depression Scale, the Pain Catastrophizing Scale, and the SF-12

| **The Hospital Anxiety and Depression Scale, the Pain Catastrophizing Scale, and the SF-12** | | | | | | | | |
| --- | --- | --- | --- | --- | --- | --- | --- | --- |
|  | Group according to pain classification | | | | | | | |
|  | CPSP  n=37 | PSP-Non  n =32 | NO-Pain  n=31 | Total  n =102 | P between  groups | CPSP x PSP-Non | CPSP x No-Pain | PSP-Non x No-Pain |
| (HAD_A) ≥ 8 | 22 (59.5%) | 15 (46.9%) | 5 (16.1%)_b_ | 42 (42.0%) | 0.001^*^ | 0.296 | <0.001^†^ | 0.009 ^†^ |
| (HAD_D) ≥ 9 | 19 (51.4%) | 12 (38.7%) | 4 (13.3%)_b_ | 35 (35.7%) | 0.005^*^ | 0.297 | 0.001^†^ | 0.024 |
| PCS | 26.3 (13.9) | 23.3 (12.0) |  | 24.9 (13.0) | 0.273 |  |  |  |
| SF-12 PCS | 31.4 (8.7) | 34.9 (9.4) | 51.2 (6.6) | 38.5 (11.9) | <0.001^*^ | 0.099 | <0.001 ^†^ | <0.001 ^†^ |
| SF-12 MCS | 38.9 (14.5) | 46.0 (13.0) | 50.8 (10.1) | 44.8 (13.6) | 0.001 ^*^ | 0.023 | <0.001^†^ | 0.169 |

Categorical variables are expressed in absolute numbers and percentages. Numerical variables are represented by mean and standard deviation.

^*^p<0.05, ^†^ p<0.0167 (pairwise comparisons Bonferroni correction for multiple comparisons).CPSP: Central Post-Stroke Pain. PSP-Non: Non-neuropathic post-stroke pain. Post-Stroke Pain. No-Pain: Stroke without pain.HAD_A: Hospital Anxiety Depression Scale subscore for anxiety. HAD_D: Hospital Anxiety Depression Scale subscore for anxiety. PCS: The Pain Catastrophizing Scale. SF-12: Short Form Health Status Questionnaire. SF-12- PCS: Physical component score. SF-12- MCS: Mental component score

Supplementary Table 8 Functional assessment: Barthel index, Modified Rankin Scale, QuickDash and Pain Disability Questionnaire

| **Functional Assessment** | | | | | | | | |
| --- | --- | --- | --- | --- | --- | --- | --- | --- |
|  | Group according to pain classification | | | | |  |  |  |
|  | CPSP  n=39 | PSP-Non  n= 32 | No-Pain  n= 31 | Total  n (%)  102 | P effects between groups | CPSP x PSP-Non | CPSP x  No-Pain | PSP-Non x No-Pain |
| Barthel index | 87.1 (20.6) | 91.7(14.6) | 98.1 (4.4) | 91.9 (15.9) | 0.013 ^*^ | 0.438 | 0.004 ^†^ | 0.028 |
| Modified Rankin Scale | | | |  | 0.013 ^Ɨ*^ | 0.44 | 0.005 ^†^ | 0.028 |
| 0 No symptoms | 0 (0%) | 0 (0%) | 5 (16.1%) | 5 (4.9%) |  |  |  |  |
| 2-Mild disability | 17(43.6%) | 13 (40.6%) | 16 (51.6%) | 46 (45.1%) |  |  |  |  |
| 2: Slight disability | 8 (20.5%) | 14 (43.8%) | 5(16.1%) | 27 (26.5%) |  |  |  |  |
| 3: Moderate disability | 7 (17.9%) | 3 (9.4%) | 5 (16.1%) | 15 (14.7%) |  |  |  |  |
| 4: Moderate to servere disability | 7 (17.9%) | 2 (6.3%) | 0 (0.0%) | 9 (8.8%) |  |  |  |  |
| Quick dash | 60.1 (26.5) | 38.1 (25.4) | 10.6 (11.6) | 38.1(30.3) | <0.001 ^*^ | 0.001^†^ | <0.001^†^ | <0.001^†^ |
| The Pain Disability Questionnaire | | | | | |  |  |  |
| Functional Status (0-90) | 54.9 (21.8) | 42.4 (18.9) |  | 49.3 (21.3) | 0.018^*^ |  |  |  |
| Psychosocial (0-60) | 32.4 (13.4) | 24.1 (14.5) |  | 28.7 (14.4) | 0.02^*^ |  |  |  |
| Total PDQ score (0-150) | 87.3 (31.1) | 66.5 (29.3) |  | 77.9 (31.8) | 0.007^*^ |  |  |  |

Categorical variables are expressed in absolute numbers and percentages. Numerical variables are represented by mean and standard deviation. *p<0.05, † p<0.0167 (pairwise comparisons Bonferroni correction for multiple comparisons). CPSP: Central Post-Stroke Pain. PSP-Non: Non-neuropathic post-stroke pain. Post-Stroke Pain. No-Pain: Stroke without pain

Supplementary Table 9: Musculoskeletal assessment Ashworth Spasticity grade, Medical Council Research and myofascial trigger points evaluation

| **Musculoskeletal assessment** | | | | | | | | |
| --- | --- | --- | --- | --- | --- | --- | --- | --- |
|  | Group according to pain classification | | | |  |  |  |  |
|  | CPSP  n= 39 | PSP-Non  n= 32 | No-Pain  n= 31 | Total  n=102 | p effects between groups | CPSP x PSP-Non^2^ | CPSP x No-Pain^2^ | PSP-Non x No- Pain^2^ |
| Ashworth Spasticity grade | | |  |  |  |  |  |  |
| Absence | 18 (46.2%) | 24 (75%) | 28(90.3%) | 70 (68.6%) | <0.001 ^*^ | 0.777 | <0.001^†^ | 0.012^†^ |
| Low to moderate (1-2) | 11 (28.2%) | 7 (21.9%) | 2 (6.5%) | 20 (19.6%) |  |  |  |  |
| Moderate to severe (3-5) | 10 (25.6%) | 1 (3.1%) | 1 (3.2%) | 12 (11.8%) |  |  |  |  |
| Paresis | n= 37 | n=32 | n=31 | n= 100 |  |  |  |  |
| Paresis grade 0 | 29.7% (11) | 25% (8) | 45.2% (14) | 33% (33) | 0.030 ^*^ | 0.181 | 0.017 | 0.151 |
| Paresis grade 1 | 29.7% (11) | 65.6% (21) | 45.2% (14) | 46% (46) |  |  |  |  |
| Paresis grade 2 | 29.7% (11) | 3.1% (1) | 9.7% (3) | 15% (15) |  |  |  |  |
| Paresis grade 3 | 10.8% (4) | 6.3% (2) | 0% (0) | 6 (6%) |  |  |  |  |
| Active myofascial trigger points | 5 (13.2%) | 24 (75.0%) | - | 29 (41.4%) | <0.001^*^ |  |  |  |

Categorical variables are expressed in absolute numbers and percentages. *p<0.05, † p<0.0167 (pairwise comparisons Bonferroni correction for multiple comparisons. Paresis grade 0 ( MRC=5) , grade 1 (MRC=4), grade 2: (MRC=2 or 3), grade 3 (MRC=1 or 0). CPSP: Central Post-Stroke PainPSP-Non: Non-neuropathic post-stroke pain. Post-Stroke Pain. No-Pain: Stroke without pain. MRC Medical Council Research

Supplementary Table 10: Myofascial Trigger Points Evaluation

| **Myofascial Trigger Points Evaluation** | | | | |
| --- | --- | --- | --- | --- |
|  | Group according to pain classification | | |  |
|  | CPSP  n=38 | PSP-Non  n= 32 | Total  n= 70 | P effects between groups |
| Active myofascial trigger points | 5 (13.2%) | 24 (75.0%) | 29 (41.4%) | <0.001^*^ |
| Temporal | 2 (5.3%) | 9 (28.1%) | 11 (15.7%) | 0.009 ^*^ |
| Masseter | 3 (7.9%) | 13 (40.6%) | 16 (22.9%) | 0.001 ^*^ |
| Scalenes | 3 (7.9%) | 15 (46.9%) | 18 (25.7%) | <0.001^*^ |
| Trapezius | 1 (2.6%) | 16 (50.0%) | 17 (24.3%) | <0.001^*^ |
| Levator scapulae | 2 (5.3%) | 12 (37.5%) | 14 (20.0%) | 0.001 ^*^ |
| Rhomboids | 0 (0.0%) | 11 (34.4%) | 11 (15.7%) | <0.001^*^ |
| Supraspinatus | 1 (2.6%) | 10 (31.3%) | 11 (15.7%) | 0.001 ^*^ |
| Pectoralis major | 0 (0.0%) | 11 (34.4%) | 11 (15.7%) | <0.00*^ß^ |
| Wrist and finger extensors | 1 (2.6%) | 10 (31.3%) | 11 (15.7%) | 0.001 ^*^ |
| First dorsal interosseous | 1 (2.6%) | 13 (40.6%) | 14 (20.0%) | <0.001^*^ |
| Quadratus lumborum | 0 (0.0%) | 15 (46.9%) | 15 (21.4%) | <0.001^*^ |
| Gluteus maximus | 1 (2.6%) | 7 (21.9%) | 8 (11.4%) | 0.02 ^*^ |
| Piriformis | 1 (2.6%) | 9 (28.1%) | 10 (14.3%) | 0.004 ^*^ |
| Vastus lateralis | 1 (2.6%) | 13 (40.6%) | 14 (20.0%) | <0.001^*^ |
| Gastrocnemius | 1 (2.6%) | 10 (31.3%) | 11 (15.7%) | 0.001 ^*^ |

Categorical variables are expressed in absolute numbers and percentages. *p<0.05. CPSP: Central Post-Stroke Pain. PSP-Non: Non-neuropathic post-stroke pain. Post-Stroke Pain. No-Pain: Stroke without pain

Supplementary Table 11: Somatossensoy gain and loss of function according to quantitative sensory testing.

|  | Group according to pain classification | | |  |
| --- | --- | --- | --- | --- |
|  | CPSP  n=38 | PSP-Non  n= 32 | Total  n= 70 | P effects between groups |
| Somatossensory gain of function | | | |  |
| DMA | 24 (61.5%)a | 2 (6.3%)b | 0 (0.0%)b | <0.001* |
| WUR | 5 (12.8%) | 6 (18.8%) | 6 (19.4%) | 0.754 |
| STCP | 3 (7.7%) | 4 (12.5%) | 3 (9.7%) | 0.914 |
| STHP | 4 (10.3%) | 3 (9.4%) | 6 (19.4%) | 0.464 |
| STMP | 8 (20.5%) | 11 (34.4%) | 7 (22.6%) | 0.429 |
| Somatossensory loss of function | | | |  |
| CDT | 18 (46.2%)a | 3 (9.4%)b | 1 (3.2%)b | <0.001* |
| WDT | 0 (0.0%) | 0 (0.0%) | 0 (0.0%) | - |
| CPT | 14 (35.9%)a | 4 (12.5%)a | 5 (16.1%)a | 0.026* |
| HPT | 15 (38.5%) | 7 (21.9%) | 7 (22.6%) | 0.508 |
| MPT | 5 (12.8%) | 4 (12.5%) | 2 (6.5%) | 0.725 |
| VDT | 12 (30.8%)a | 6 (18.8%)a,b | 1 (3.2%)b | 0.005* |
| MDT | 12 (30.8%)a | 2 (6.3%)a | 3 (9.7%)a,b | 0.030* |

Patients were classified with normal or abnormal parameters on quantitative sensory evaluation according to values proposed by Roke et al, 2006^89^.Categorical variables are expressed in absolute numbers and percentages. *p<0.05. Numbers followed by different letters are statistically different in subgroup analysis with Bonferroni correction (p<0.0167) CPSP: Central Post-Stroke Pain. PSP-Non: Non-neuropathic post-stroke pain. Post-Stroke Pain. No-Pain: Stroke without pain. CDT: Cold detection threshold. WDT: Warm detection threshold. MDT: Mechanical detection threshold. CPT: Cold pain threshold. HPT: Heat pain threshold. MPT: Mechanical pain threshold. VDT: Vibration detection threshold. STCP: suprathreshold cold pain ̶ pain referred according to the Numeric Pain Rating Scale (NRS) after suprathreshold cold pain stimulus. STHP: suprathreshold heat pain ̶ NRS after suprathreshold heat pain stimulus. STMP: NRS after suprathreshold mechanical pain stimulus. WUR wind up ratio:

| **QST modality** | **Odds ratio** | **IC 95% inf** | **IC95% sup** | **P** |
| --- | --- | --- | --- | --- |
| Temporal summation | 4.4 | 1.9 | 10.5 | 0.001 |
| Cold hypoesthesia | 12 | 3.8 | 41.6 | <0.001 |
| Cold hypoalgesia | 3.4 | 1.28 | 8.8 | 0.011 |
| Mechanical hypoesthesia | 5.2 | 1.6 | 16.1 | 0.03 |
| Hypopallesthesia | 3.6 | 1.3 | 10.0 | 0.013 |
| STT | 9.1 | 3.1 | 26.5 | <0.001 |
| LMN | 5.0 | 2.1 | 12 | <0.001 |

Supplementary Table 12: Odds ratio of neuropathic pain in quantitative sensory testing modalities

QST: Quantitative sensory testing. STT spinothalmic tract impairment ( cold detection, cold pain, warm detection, heat pain or mechanical pain thresholds impairment. LMN leminiscal tract impairment ( mechanical or vibratory thresholds) The other QST modalities did not evidence statistical significance.

Supplementary Table 13 Conditioned Pain Modulation (CPM) according to pain groups

|  | CPSP  n=24 | PSP-Non  n=30 | No-pain  n=30 | Comparisons between groups | CPSP x PSP-Non | CPSP x No-Pain | PSP-Non x No-Pain |
| --- | --- | --- | --- | --- | --- | --- | --- |
| Unconditioned stimuli | 74.5 (56.7-92.7) | 80.0 (63.7-92.0) | 80.0 (58.5-91.2) |  |  |  |  |
| Conditioned stimuli | 70.0 (42.2-86.0) | 72.0 (47.5-92.0) | 53.0 (27.2-81.2) |  |  |  |  |
| CPM | 1.5 (-2.7-22.5) | 0.0 (-5.0-16.0) | 10.0 (0-40) | 0.047 ^*^ | 0.669 | 0.093 | 0.016 ^†^ |

Unconditioned and conditioned stimuli were measured through the numerical rating pain scale. Numerical variables are represented by median and p25 and p75^.^ Level. *p<0.05, † p<0.0167 (pairwise comparisons Bonferroni correction for multiple comparisons. CPSP: Central Post-Stroke Pain. PSP-Non: Non-neuropathic post-stroke pain
